# Shining light on drug discovery: optogenetic screening for TopBP1 biomolecular condensate inhibitors

**DOI:** 10.1093/narcan/zcaf041

**Published:** 2025-11-03

**Authors:** Laura Morano, Nadia Vie, Adam Aissanou, Dana Hodroj, Véronique Garambois, Alexandra Fauvre, Alexy Promonet, Tom Egger, Benoît Bordignon, Cédric Hassen-Khodja, Solène Fiachetti, Jihane Basbous, Céline Gongora, Angelos Constantinou

**Affiliations:** IGH, Univ Montpellier, CNRS, Montpellier 3436, France; Institut de Recherche en Cancérologie de Montpellier, INSERM, CNRS, Univ Montpellier, Montpellier 34298, France; Institut de Recherche en Cancérologie de Montpellier, INSERM, CNRS, Univ Montpellier, Montpellier 34298, France; IGH, Univ Montpellier, CNRS, Montpellier 3436, France; Institut de Recherche en Cancérologie de Montpellier, INSERM, CNRS, Univ Montpellier, Montpellier 34298, France; Institut de Recherche en Cancérologie de Montpellier, INSERM, CNRS, Univ Montpellier, Montpellier 34298, France; IGH, Univ Montpellier, CNRS, Montpellier 3436, France; IGH, Univ Montpellier, CNRS, Montpellier 3436, France; Montpellier Ressources Imagerie BioCampus, Univ Montpellier, CNRS, INSERM, Montpellier 34298, France; Montpellier Ressources Imagerie BioCampus, Univ Montpellier, CNRS, INSERM, Montpellier 34298, France; IGH, Univ Montpellier, CNRS, Montpellier 3436, France; IGH, Univ Montpellier, CNRS, Montpellier 3436, France; Institut de Recherche en Cancérologie de Montpellier, INSERM, CNRS, Univ Montpellier, Montpellier 34298, France; IGH, Univ Montpellier, CNRS, Montpellier 3436, France

## Abstract

Human topoisomerase IIβ binding protein 1 (TopBP1) is a scaffold protein involved in DNA replication initiation, DNA repair, transcription regulation, and checkpoint activation. TopBP1 forms nuclear condensates that act as a molecular switch to amplify ATR activity and promote the activation of the checkpoint effector kinase Chk1. In cancer cells, ATR activity is crucial to tolerate the intrinsically high level of DNA lesions and obstacles that block replication fork progression. Thus, ATR inhibitors are currently tested in clinical trials, often in combination with chemotherapy drugs. However, resistance and toxicity are still major issues. The weak interactions that hold TopBP1 condensates together are highly sensitive to changes in the cellular milieu, suggesting that small molecules may alter the formation of TopBP1 condensates. Here, we developed a high-throughput screening system to identify TopBP1 condensation modulators. This system allowed us to identify FDA-approved drugs, including thimerosal and quinacrine, that inhibit TopBP1 condensation and block the activation of ATR/Chk1 signaling. Mechanistically, quinacrine impaired TopBP1’s ability to associate with chromatin, thereby interfering with its capacity to form condensates. Furthermore, quinacrine enhanced the therapeutic efficacy of 5-fluorouracil and irinotecan, components of the clinically used FOLFIRI regimen in a mouse model of peritoneal carcinomatosis from colorectal cancer.

## Graphical Abstract

**Graphical Abstract:**
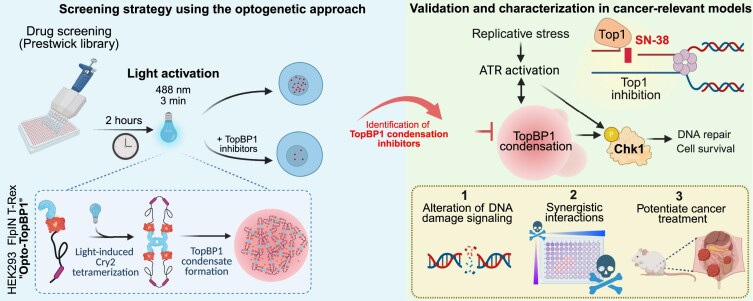

## Introduction

Every day, thousands of DNA lesions occur in cells because of internal and environmental factors. Cancer cells display higher levels of genotoxic stress than normal cells due to their disturbed metabolism and rapid proliferation [1–3]. Cells have developed DNA damage response pathways to counteract the harmful effects of DNA lesions. These pathways detect and signal DNA damage and orchestrate its repair while synchronizing with essential cellular processes (DNA replication, transcription, and chromatin remodeling) [4, 5]. In cancer treatment, alongside conventional approaches such as radiotherapy and chemotherapy that induce DNA damage, innovative treatment strategies are emerging with the aim of increasing the cancer cell sensitivity to genotoxic stress by targeting DNA damage signaling and repair mechanisms [1, 6]. For instance, the ATR signaling pathway plays a pivotal role in activating cell cycle checkpoints [7–9], facilitating DNA repair [10], and regulating DNA replication [11] and the nucleotide pool [12–15]. Given its critical role in allowing cell proliferation in the presence of high levels of genotoxic stress, clinical trials are investigating the efficacy of ATR inhibitors in cancer [16]. However, challenges persist because kinase inhibitors are very toxic and often drive the emergence of drug resistance through mutations, necessitating alternative therapeutic strategies [17].

Topoisomerase IIβ binding protein 1 (TopBP1), a scaffold protein composed of nine BRCA1 carboxyl terminal (BRCT) protein–protein interaction motifs [18, 19], is the main ATR activator in the S phase of the cell cycle through its intrinsically disordered ATR-activation domain positioned between BRCT6 and BRCT7-8 [18, 20]. We recently showed that TopBP1 activates the ATR signaling pathway through the assembly of biomolecular condensates that can be observed as nuclear foci using fluorescence microscopy [21]. These condensates are specialized compartments within cells that concentrate proteins and nucleic acids without any membrane [22, 23]. Biomolecular condensates organize a variety of biological processes, and their deregulation has been implicated in many diseases, including cancer [24]. In the presence of replication stress, single-stranded DNA is recognized by RPA [25] that will recruit the ATRIP–ATR complex [26] and the 9–1–1 complex, through the RAD17–RFC2-5 complex, to stabilize the junction between single- and double-stranded DNAs. TopBP1 is recruited on RAD9, which is part of the 9–1–1 complex [27, 28], a process facilitated by MRE11 [29]. Then, TopBP1 is in the proximity of ATR and can be phosphorylated by the basal kinase activity of ATR to trigger the assembly of TopBP1 condensates [21]. This acts as a molecular switch that amplifies ATR activity up to the level required for signal transduction by the checkpoint effector kinase Chk1 [21].

A new alternative to the traditional approach of targeting specific proteins with small-molecule inhibitors is to focus on biomolecular condensates for drug discovery [24]. As condensate formation is driven by multivalent protein scaffolds, such as TopBP1 [22, 23], one potential approach to modify condensate properties and functions is to interfere with essential protein–protein or protein–nucleic acid interactions.

To this end, we previously described a framework to identify TopBP1 condensate inhibitors to downregulate ATR signaling and consequently, enhance chemotherapy drug effectiveness in colon cancer cells [30]. In the present study, we applied the same framework described by Morano *et al.*, which includes a high-throughput optogenetic screening platform to discover novel TopBP1 condensation modulators and their *in vitro*/*in vivo* validation. We focused on quinacrine, an antimalarial drug and a candidate drug for the treatment of refractory cancers [31]. Quinacrine targets different pathways, but the mechanisms underlying its selective targeting of cancer cells remain poorly understood. Here, we found that quinacrine blocks the formation of TopBP1 condensates, inhibits ATR signaling, and improves the efficacy of 5-fluorouracil and irinotecan (mimicking the FOLFIRI combination used in the clinic) in a mouse model of peritoneal carcinomatosis from colorectal cancer. The data support the feasibility of targeting condensates formed in response to DNA damage for improving chemotherapy-based cancer treatments.

## Materials and methods

### Cell culture

Cells were grown in a 37°C humidified incubator with 5% CO_2_. Flp-In™ 293 T-REx cells were obtained from Thermo Fisher and cultured in Dulbecco’s modified Eagle medium (DMEM)-GlutaMAX medium (Merck–Sigma–Aldrich, #D5796) supplemented with 10% heat-inactivated fetal bovine serum (FBS). For the generation of stable cell lines that express TopBP1 fused to the photoreceptor cryptochrome 2 (Cry2) and mCherry (optoTopBP1), please refer to [21]. The HCT116 human colorectal cancer cell line (ATCC, #CCL247), LNCaP human prostate cancer cell line (CRL-1740), and CT26 murine colorectal cancer cell line were cultured in RPMI-1640 (Sigma, #R8758; 500 ml) with 10% heat-inactivated FBS. The SN-38-resistant HCT116-SN6 and HCT116-SN50 clones were obtained in the laboratory as described previously [32]. Briefly, SN-38-sensitive parental HCT116 cells were grown in the presence of 10 and 15 nM SN-38 to obtain the low-level resistant HCT116-SN6 clone and the high-level resistant HCT116-SN50 clone, respectively. Then, HCT116-SN6 and HCT116-SN50 cells were grown in RPMI-1640 (Sigma #R8758; 500 ml) supplemented with 10% heat-inactivated FBS. Cells were regularly tested for mycoplasma infection.

### Drugs and treatments

The Prestwick Chemical Library (Illkirch-Graffenstaden, France) containing 1520 FDA-approved molecules was tested in the high-throughput optogenetic screen (see below). SN-38 (active metabolite of irinotecan; #2684 Tocris) was diluted in DMSO (Dimethyl Sulfoxide) to 10 mM and stored at −20°C. 5-Fluorouracil (5-FU) was from the ICM pharmacy (Fluorouracil ACCORD Cip: 3400957518288) and was diluted in phosphate-buffered saline (PBS) to the final concentration of 380 mM for 5-FU (stored at room temperature). FOLFIRI concentration range and IC50 are indicated in Supplementary Tables S2, S3, and S4. Quinacrine and thimerosal were diluted in pure water to 6 and 21 mM, respectively, and stored at −80°C. For the 2-h incubation, drugs were diluted in RPMI (10% FBS) to 300 nM for SN-38, 40 µM for quinacrine (Sigma #Q3251), and 20 µM for thimerosal (Sigma #T5125).

### High-throughput optogenetic screening at the MRI facility platform

OptoTopBP1-expressing Flp-In 293 T-Rex cells were seeded in black clear-bottom 384-well plates (Phenoplate, Revvity) at a density of 4000 cells/well in DMEM supplemented with 10% FBS and 2 μg/ml doxycycline using a Multidrop Combi Dispenser (Thermo Scientific™) and allowed to attach overnight at 37°C, 5% CO_2_ for 72 h. Then, cells were treated with the Prestwick Chemical Library of USA Food and Drug Administration (FDA)-approved drugs at a concentration of 10 μM using a Tecan EVO^®^200 robotic liquid handling system (Tecan) and incubated for 2h at 37°C, 5% CO_2_ in an automated incubator (Cytomat C6001, Thermo Scientific™) integrated within the Tecan robot. From the 10 mM stock solutions in DMSO, each compound was diluted to 2 mM in daughter plates (intermediated dilution in DMSO) before addition to the wells by automated pipetting at a final concentration of 10 μM (0.5% DMSO). After incubation, cells were exposed to cycling pulses (4 s ON, 10 s OFF) of 488 nm blue light for 3 min. Cells were fixed immediately in 8% paraformaldehyde (Euromedex, #15710-S) diluted in PBS for 20 min. Cells were automatically rinsed with PBS twice, and nuclei were counterstained with 0.5 μg/ml Hoechst 33342 (Thermo Fisher Scientific, #62 249), 100 µl/well in PBS, using an automated plate washer (Hydrospeed, Tecan). High-content imaging was performed with the Opera Phenix™ High Content Screening System confocal (Revvity). Fifteen fields of view per well were imaged with a 63× water objective in confocal mode. Then, image analysis and quantification of output parameters were obtained on the fly with Harmony^®^ High-content analysis software (v4.9, Revvity). Briefly, nuclei were segmented with the Hoechst signal and nuclear spots (condensates) were counted with the mCherry signal. Data were then analyzed using a specific statistical methodology (see below). The full list of compounds screened in the Prestwick library with replicate counts, individual scores, average score, and hit assignment is described in Supplementary Table S5.

### Screening data normalization and statistical analysis

Data from HCS readout were analyzed using R programming language (RStudio software) and assessed for robustness and reproducibility by calculating the *Z*’-factor [33] using the following equation:


\begin{eqnarray*}
Z^{\prime} \hbox{-} \text{factor} = 1 - \frac{{3\left( {{{\sigma }_p} + {{\sigma }_n}} \right)}}{{\left| {{{\mu }_p} - {{\mu }_n}} \right|}},
\end{eqnarray*}

where ${{\mu }_p}$ and ${{\sigma }_p}$ are the median and median absolute deviation values of the positive control and ${{\mu }_n}$ and ${{\sigma }_n}$ are those of the negative control.

Normalization and hit identification were performed separately for each plate [34]. A median-based normalization was applied to correct for plate effects by using the median value across wells that were not annotated as controls.

Subsequently, a *z*-score was assigned to each molecule using the following equation (*z*-scores of the three replicates were averaged):


\begin{eqnarray*}
z \hbox{-} \text{score} = \frac{{x^{\prime}ki - \text{median}\left( {x^{\prime}i} \right)}}{{\text{mad}\left( {x^{\prime}i} \right)}},
\end{eqnarray*}

where *x*’ is the normalized values, *k*th well and *i*th plate.

### Western blotting

Whole-cell extracts were obtained by lysing cells in RIPA buffer [50 mM Tris, 150 mM NaCl, 1% NP-40, 1% deoxycholate, 0.1% sodium dodecyl sulfate (SDS), pH 8] on ice for 30 min. After centrifugation, supernatants were collected, and the amount of protein was quantified using the Pierce™ BCA Protein Assay Kit (Thermo Fisher, # 23225). Laemmli buffer was added, and proteins were boiled at 95°C for 5 min. Forty micrograms of each protein sample was resolved on precast sodium dodecyl sulfate–polyacrylamide gel electrophoresis (4%–15%, 7.5%, and 10%) and transferred to nitrocellulose membranes using the Bio-Rad Trans-Blot Turbo transfer device. Membranes were blocked with 5% nonfat milk diluted in TBS/0.1% Tween 20 (TBS-T) and incubated with primary antibodies at 4°C overnight. The antibodies used were against phosphorylated Chk1 (Ser345) (Cell Signaling, #2348), Chk1 (Santa Cruz Biotechnology, #sc8408), TopBP1 (Euromedex, #A300-111A or Santa Cruz, #sc-271043), α-tubulin (Sigma, #T5168), H3 (Cell Signaling, #4499S), vinculin (Merck, #V9131), γH2AX (Cell Signaling, #9718S; Millipore, #05-636), phosphorylated RPA (Ser33) (Abcam, #ab2118877), RPA (Abcam, #ab2175), H3K4me3 (Abcam, #ab8580), H3K9Ac (Sigma, #07-352), and H3K9me3 (Abcam, #ab8898). Then, membranes were incubated with anti-mouse (Cell Signaling, #7076S) or anti-rabbit HRP-conjugated (Cell Signaling, #7074S) secondary antibodies at room temperature (RT) for 1 h, followed by ECL Clarity (BIO-RAD, #170-5061) or ECL select (BIO-RAD, #1 705062), according to the manufacturer’s instructions.

### Immunofluorescence

Cells were grown on 1.5H glass coverslips, and after incubation with the indicated drugs for 2 h, the soluble cell fraction was eliminated by a pre-extraction step in Cytoskeleton buffer (1× PBS, 0.2% Triton X-100) at 4°C for 60 s. Then, cells were fixed with 4% paraformaldehyde in PBS at RT for 15 min. Blocking was performed by incubation with the blocking solution [PBS/5% bovine serum albumin (BSA)] for 30 min. For immunofluorescence staining, anti-TopBP1 (Santa Cruz, #sc-271043, diluted at 1/100) or anti-PML (Santa Cruz, #sc-966, diluted at 1/300) primary antibodies and the anti-mouse secondary antibody coupled to fluorochrome (Invitrogen, #A-11011, diluted at 1/500) were diluted in blocking solution and incubated for 1 h and 45 min, respectively. Washes between incubation steps were performed with PBS/0.1% Tween 20 at RT (3 × 5 min). Hoechst was used for DNA staining, and coverslips were stored at RT after mounting with Prolong Gold antifade reagent (Invitrogen, #P36930). Images were taken with Zeiss AxioImager Apotome (Z2) using a 63× objective at the Montpellier Ressources Imagerie (MRI) facility.

### Nuclear extracts

Nuclear extracts were prepared using Dignam’s method as previously described [35, 36]. This well-established protocol yields functionally active nuclear proteins and is widely used for *in vitro* studies of transcription and DNA damage response. It closely mimics the native biochemical environment of the nucleus, allowing for controlled mechanistic analyses of nuclear processes that are otherwise difficult to study in intact cells. HeLa S3 or HCT116 cells were grown to ≤80% confluence, collected by scraping and centrifugation [200 × *g* for 3 min at 4°C], and washed twice in PBS. The cell pellet was suspended in 5× packed cell volume of hypotonic buffer A (10 mM Hepes–KOH, pH 7.9, 10 mM KCl, 1.5 mM MgCl_2_, 0.5 mM dithiothreitol (DTT), and 0.5 mM Phenylmethylsulfonyl fluoride (PMSF)), supplemented with a cocktail of protease inhibitors (cOmplete, ethylenediaminetetraacetic acid (EDTA) free; Roche) and phosphatase inhibitors (Thermo Fisher Scientific), and incubated on ice for 5 min. Next, the cells were spun down at 500 × *g* for 5 min, suspended in 2× packed cell volume of buffer A, and lysed by Dounce homogenization using a tight-fitting pestle. Nuclei were collected by centrifugation at 4000 × *g* for 5 min at 4°C and extracted in one nuclei pellet volume of buffer C (20 mM Hepes–KOH, pH 7.9, 600 mM KCl, 1.5 mM MgCl_2_, 0.2 mM EDTA, 25% glycerol, 0.5 mM DTT, and 0.5 mM PMSF) supplemented with cocktails of protease and phosphatase inhibitors, and mixed on a rotating wheel at 4°C for 30 min. Nuclear extracts (supernatants) were recovered by centrifugation (16 000 × *g* for 15 min at 4°C) and dialyzed using Slide-A-Lyzer Dialysis Cassettes (3500-Da protein molecular weight cutoff; Thermo Fisher Scientific) against buffer D (20 mM Hepes–KOH, pH 7.9, 100 mM KCl, 0.2 mM EDTA, 20% glycerol, 0.5 mM DTT, and 0.5 mM PMSF). Dialyzed nuclear extracts were centrifuged (100 000 × *g* for 30 min at 4°C) to eliminate residual precipitates. The protein concentration of the clear supernatant was determined using Bradford’s estimation method, and aliquots were snap-frozen and stored at −80°C.

### Cytometric assays

After incubation with quinacrine (40 µM) and/or SN-38 (300 nM) for 2 h, cells were trypsinized and collected into pre-chilled 1.5-ml Eppendorf tubes, followed by centrifugation at 400 × *g*, 4°C for 3 min. Pellets were washed with 500 µl of PBS and centrifuged at 400 × *g*, 4°C for 3 min. Then, pellets were resuspended in 100 µl of PBS/0.2% Triton X-100 and incubated on ice for 10 min. Next, 1 ml of PBS-B (1 mg/ml BSA in PBS) was added to the cell suspensions that were centrifuged at 550 × *g* at 4°C for 3 min. Cells were fixed with 100 µl of 4% paraformaldehyde in PBS at RT for 15 min. Then, 500 µl of 1× BD Perm/Wash buffer (BD Biosciences # 554723, diluted in H_2_O) was added to the fixed cells that were centrifuged at 700 × *g* for 3 min. For immunostaining, primary antibodies against phosphorylated RPA (Ser33) and γH2AX (see references in the “Western blotting” section) were diluted in 1× Perm/Wash buffer. Cell pellets were resuspended in 50 µl of primary antibody solution and incubated at 4°C under rotation overnight. Subsequently, 500 µl of Perm/Wash buffer was added, and cells were centrifuged at 700 × *g* for 3 min. Then, 50 µl of diluted secondary antibodies (anti-mouse Alexa Fluor 488 Invitrogen #A-11001; anti-rabbit Alexa Fluor 647 Invitrogen # A-21245) was added, and cells were incubated at RT in the dark for 45 min. After centrifugation (3 min at 700 × *g*), cells were resuspended in 500 µl of analysis Buffer (250 μg/ml RNase, 0.5 μg/ml DAPI (4′,6-diamidino-2-phenylindole), and 1 mg/ml BSA in PBS) and incubated at 37°C for 20–30 min. Cells were analyzed with a Gallios flow cytometer (Beckman Coulter) at the MRI facility.

### Cell fractionation

The protocol used was described by Wysocka *et al.* [37]. Briefly, cells were collected, washed with PBS, and resuspended in buffer A [10 mM HEPES (pH 7.9), 10 mM KCl, 1.5 mM MgCl_2_, 0.34 M sucrose, 10% glycerol, 1 mM DTT, and protease inhibitors (Roche)]. After the addition of Triton X-100 (0.1% final concentration), cells were incubated on ice for 5 min, and nuclei were collected by centrifugation (1300 × *g*, 4°C, 5 min). Supernatants (cytosolic fraction) were clarified by high-speed centrifugation (20 000 × *g*, 4°C, 5 min), and supernatants (cytosolic fraction) were collected. Then, nuclei were washed once in buffer A and lysed in buffer B [3 mM EDTA, 0.2 mM EGTA, 1 mM DTT and protease inhibitor (Roche)] for 30 min, and the insoluble chromatin and soluble fractions (nucleosolic fraction) were separated by centrifugation (17 000 × *g*, 4°C, 5 min). The insoluble chromatin fraction was washed twice with buffer B, resuspended in SDS–Laemmli buffer, and boiled for 10 min. Western blotting was performed using the ECL procedure according to the manufacturer’s instructions (Amersham Bioscience, Inc.).

### Chk1 phosphorylation immunofluorescent detection with the Celigo Imaging Cytometer

HCT116 cells were seeded in a 384-well plate (Greiner, #78 109) at 1300 cells/well in 50 µl of medium. Forty-eight hours after seeding, 50 µl of medium containing the drugs of interest was added for 2 h. Positive hits from the Prestwick Chemical Library were combined with SN-38 at 300 nM, a concentration that induces the phosphorylation of Chk1 (pChk1) (data not shown) at a level that allows the detection of its decrease in the presence of positive hits. After the 2-h treatment, the medium was removed, and cells were fixed and permeabilized with 4% paraformaldehyde (Euromedex, #15710-S)/0.1% Triton X-100/PBS at RT for 10 min. Wells were rinsed with PBS and saturated with 3% BSA/PBS at RT for 1 h. The primary antibody against phosphorylated Chk1 (Cell Signaling Technology, #2348L), diluted at 1/50 in 1% BSA/PBS, was added to each well and incubated at 4°C under low agitation overnight. Wells were washed (3 × 5 min) at RT with 0.05% Tween 20/PBS. Then, the secondary fluorochrome-conjugated antibody (anti-rabbit-AF568 from Invitrogen, #A11011) diluted at 1/1000 in 1% BSA/PBS was added at RT for 45 min. Wells were washed (3 × 5 min) with 0.05% Tween 20/PBS at RT, and nuclei were stained with Hoechst (#33342, 1 µg/ml final). Images were acquired with a Celigo Imaging Cytometer (Nexcelom Bioscience) at the MRI facility using the “Target 1 + Mask” channel and analyzed with the Celigo satellite software to calculate the percentage of pChk1-positive cells, the mean pChk1 signal intensity per well, and the total cell count.

### Cell growth inhibition assay

Cell growth was evaluated using the sulforhodamine B (SRB) assay, as described by Skehan *et al.* [38]. Briefly, 500 cells/well were seeded in 96-well plates. After 24 h, drugs were added in serial dilution. Cells were incubated for 96 h, followed by fixation in 10% trichloroacetic acid solution (SIGMA, #T9159) and staining with 0.4% SRB (SIGMA, #S9012) in 1% acetic acid (FLUKA, #33 209). Plates were washed three times with 1% acetic acid, and fixed SRB was dissolved with 10 mmol/l Tris-Base solution (Trizma^®^base SIGMA). The absorbance was read at 560 nm using a PHERAstar FS plate reader (BMG LABTECH). The IC50 was determined graphically on the cell growth curves.

### Spheroid assay

Fifty cells/well were seeded in ultra-low attachment 96-well round-bottomed plates (Corning B.V. Life Sciences) and allowed to form spheroids at 37°C, 5% CO_2_, for 3 days. Then, spheroids were incubated with a concentration range of quinacrine or thimerosal and/or of chemotherapy drug (SN-38 or 5-FU) for 7 days. Then, dead cells were stained with propidium iodide, and images were acquired with a Celigo Imaging Cytometer (Nexcelom Bioscience) using the channels “Tumorosphere” and “BrightField + Mask”. Cell viability was quantified using the ATP measurement luminescent signal with CellTiterGlo for 3D cultures (Promega, #G9683) according to the manufacturer’s instructions. Luminescence was measured in white 96-well plates using the PHERAstar FS plate reader (BMG LABTECH).

### Synergy matrix

The percentage of living cells after incubation with each drug alone or in combination was calculated and normalized to untreated cells. Then, using a script in the “R” software based on the effect of each molecule alone (Bliss and Lehàr equation) [39], a synergy matrix was generated. The number associated with each combination, when positive, indicates the part of the observed effect due to the synergy; when negative, it indicates an antagonism between molecules. On the basis of the associated number, each combination is defined by a color that indicates the type of effect observed. Synergy is associated with the color red, additivity with black, and antagonism with green.

### *In vitro* cytotoxicity assays

To characterize the cytostatic and/or cytotoxic effect of quinacrine and FOLFIRI, alone and in combination in HCT116 and CT26 cells, after incubation, cells were stained with propidium iodide/Hoechst. Cells were seeded at 500 cells/well in black flat-bottom 96-well plates, cultured for 24 h, and then the quinacrine-FOLFIRI combination was added for 96 h. Then, propidium iodide (P4864 SIGMA) and Hoechst 33 342 (Thermofisher Scientific) were added at 1 and 5 µg/ml, respectively, at 37°C for 30 min. Cells were counted based on the respective fluorescence signals with a Celigo Imaging Cytometer (Nexcelom Bioscience) using the “Expression analysis—Cell Viability − Dead + Total” application. The number of live cells was calculated by subtracting the number of dead cells from the number of total cells that were evaluated by Hoechst staining. The percentages of live and dead cells as a function of the drug concentrations allowed determining the drug cytotoxic or cytostatic profile in the treated cell line, as described previously [40].

### *In vivo* studies

#### Colorectal cancer cell grafts

One hundred thousand CT26-luc tumor cells were injected into the intraperitoneal cavity of 5-week-old female Balb/c mice (Charles River Laboratories; *n* = 6). Tumors were detected by bioluminescence signal analysis after injection of 0.2 ml Luciferine (IVISbrite D-Luciferin Potassium Salt Bioluminescent Substrate XenoLight PerkinElmer^®^) and were measured weekly with the IVIS Lumina II *In Vivo* Imaging System (PerkinElmer^®^). Signals were quantified with the Living Image 4.5.2 software (PerkinElmer). Mice were euthanized when the tumor bioluminescence signal reached a threshold of 10^10^ photons. Ethical approval was obtained by the local ethics committee [Ethics Committee approved by the French Ministry, animal facility approval D 34-172-27, personal approval (Celine Gongora) 34.142, and protocol approval APAFIS#25 332].

#### Tumor treatment

Mice were treated from day 7 after tumor injection for one month with: (i) 0.2 ml of 0.9% sodium chloride solution by intraperitoneal injection twice per week (non-treated group); (ii) 0.15 ml of 50 mg/kg quinacrine in 5% sucrose water *per os* three times per week; (iii) 0.2 ml of 25 mg/kg 5-FU and 25 mg/kg irinotecan and 50 mg/kg leucovorin (FOLFIRI) in 0.9% NaCl solution by intraperitoneal injection twice per week; and (iv) 0.2 ml of 25 mg/kg 5-FU and 25 mg/kg irinotecan and 50 mg/kg leucovorin (FOLFIRI) in 0.9% NaCl solution by intraperitoneal injection twice a week + 0.15 ml of 50 mg/kg quinacrine in 5% sucrose water *per os* three times per week.

Tumor spread in mice was determined at treatment end as the peritoneal carcinomatosis index (PCI), based on the exploration of 13 standard abdominal regions during animal dissection. The scoring combines the number of abdominal regions containing tumor nodules and their size [41].

#### Software tools

R Studio was used for drug combination analyses, and Living Image 4.5.2 (PerkinElmer) for tumor bioluminescence signal quantification. CellProfiler 4.2.8 pipelines were used for foci quantifications, as previously described [42]. SuperPlotsOfData [43] was used to overlay data from different biological replicates, as previously described [40]. Fiji was used to quantify band intensity in western blot experiments. GraphPad 10.2.1 was used for generating the graphs and performing the statistical analysis. Kaluza was used for cytometry experiments. ImageLab 5.2.1 was used for western blot images and Adobe Illustrator CS6 for figures.

### Statistical analysis

For immunofluorescence foci quantification, one-way ANOVA tests were used to assess statistical differences between biological conditions, unless stated otherwise. Šídák’s multiple comparison test was applied for post hoc pairwise group comparisons. All statistical analyses were performed with GraphPad 10.2.1.

## Results

### Optogenetics as a screening tool to identify inhibitors of TopBP1 biomolecular condensates

**Figure 1.**
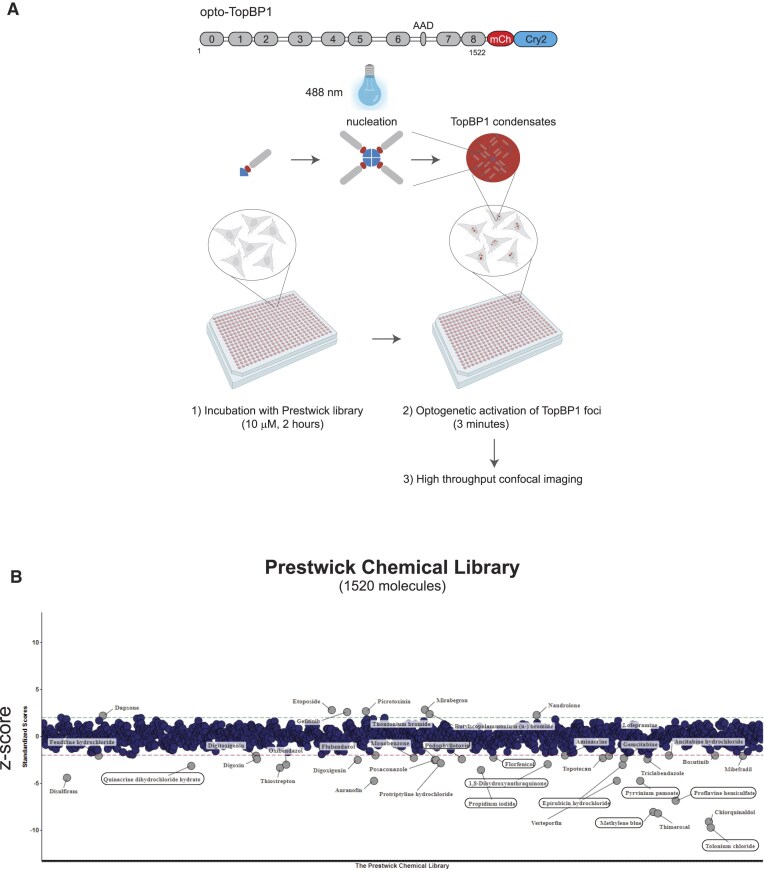
High-throughput optogenetic screening of TopBP1 condensation inhibitors. (**A**) Schematic representation of the high-throughput screening system. HEK293 cells were stably transfected with TopBP1 fused to mCherry and the light-sensitive cryptochrome 2 (Cry2) at its C-terminus, the expression of which can be induced by incubation with doxycycline. In these cells, exposure to blue light (4 s on, 10 s off) for 3 min allows inducing TopBP1 condensation without any DNA damage. Cells were grown in 384-well plates and incubated with 10 µM of Prestwick Chemical Library/well for 2 h before optogenetic activation of TopBP1 condensate formation. (**B**) Graphic representation of the Prestwick Chemical Library screening results. Drugs with a *z*-score below −2 and above 2 are considered to be TopBP1 condensation inhibitors and activators, respectively. The normalized values were calculated by subtracting the median value of each measurement (over all plates) and dividing the results by the overall mean absolute deviation (screening performed in triplicate). The potential TopBP1 condensate inhibitors that act on the DNA structure are circled.

We previously developed an optogenetic system for screening molecules that disrupt the formation of TopBP1 biomolecular condensates [30]. We used the FlpIN HEK293 cell line that expresses TopBP1 fused to the photoreceptor cryptochrome 2 (Cry2) and mCherry in a doxycycline-induced manner (optoTopBP1-HEK293 cells). Upon exposure to 488 nm light, Cry2 forms tetramers and then nucleates the assembly of TopBP1 condensates [21] (Fig. 1A). The mCherry tag allows the direct visualization of TopBP1 by fluorescence microscopy. The advantage of this optogenetic system is that TopBP1 condensates are formed on demand, within minutes, in the absence of DNA damaging agents that can induce confounding effects after prolonged treatments.

In this study, we screened the Prestwick Chemical Library of USA FDA-approved drugs in a high-throughput format. This library includes 1520 chemically diverse therapeutic compounds. We seeded optoTopBP1-HEK293 cells in 384-well plates, pre-incubated them with the compound library at a final concentration of 10  µM per well for 2 h, and then exposed the cells to 488 nm light for 3 min to induce the formation of TopBP1 condensates (Fig. 1A). Next, we fixed the cells for high-throughput quantification of TopBP1 condensates to identify the molecules that inhibit this process. This allowed us to identify 31 potential inhibitors (Fig. 1B and Supplementary Table S1), among which approximately half were DNA intercalators or DNA-binding molecules (Table 1). As chromatin can provide a surface for the condensation of DNA-binding proteins [44, 45], we hypothesized that this class of molecules could interfere with TopBP1 condensation by modifying the chromatin architecture. To investigate the phenotypic consequences of TopBP1 condensation inhibition by such small molecules, we focused on thimerosal and quinacrine. Thimerosal is an organic mercury compound that reacts with thiols and inhibits the growth of cancer cells [46], including the colon cancer cell line HCT116 [47]. Quinacrine belongs to the family of acridine compounds that includes proflavine, also identified in this screen. As it has been used for many diseases, including autoimmune diseases, and as a prophylactic treatment against malaria during World War II [31, 48], many data are available on quinacrine pharmacokinetics and safety [48]. Moreover, as quinacrine is considered for the treatment of refractory cancers [31], we hypothesized that it may improve the response of colon cancer cells to conventional therapeutic treatments.

**Table 1.** Hit molecules that affect DNA integrity identified in the optogenetic-based screen

Molecule	Effect on DNA
Tolonium chloride	Intercalates into calf thymus DNA + electrostatic [45, 46]
Methylene blue	DNA intercalator + electrostatic [47, 48]
Proflavine hemisulfate	DNA intercalator [49]
Pyrvinium pamoate	Binds to DNA [50]
Propidium iodide	DNA intercalator, binds also to core histones [51]
Quinacrine	DNA intercalator [47, 52]
Epirubicin hydrochloride	Anthracycline, DNA intercalator [53]
1,8-Dihydroxyanthraquinone	DNA intercalator [54]
Podophyllotoxin	Topoisomerase II inhibitor [55]
Dogoxin	Topoisomerase I and II inhibitor [56]
Gemcitabine	Deoxycytidine analogue [57]
Topotecan	Topoisomerase I inhibitor [58]
Florfenicol	Binds to the minor groove of DNA [59]
Ancitabine hydrochloride	Metabolized to cytarabine incorporated instead of cytidine [60]

### Quinacrine and thimerosal inhibit the formation of endogenous TopBP1 condensates

**Figure 2.**
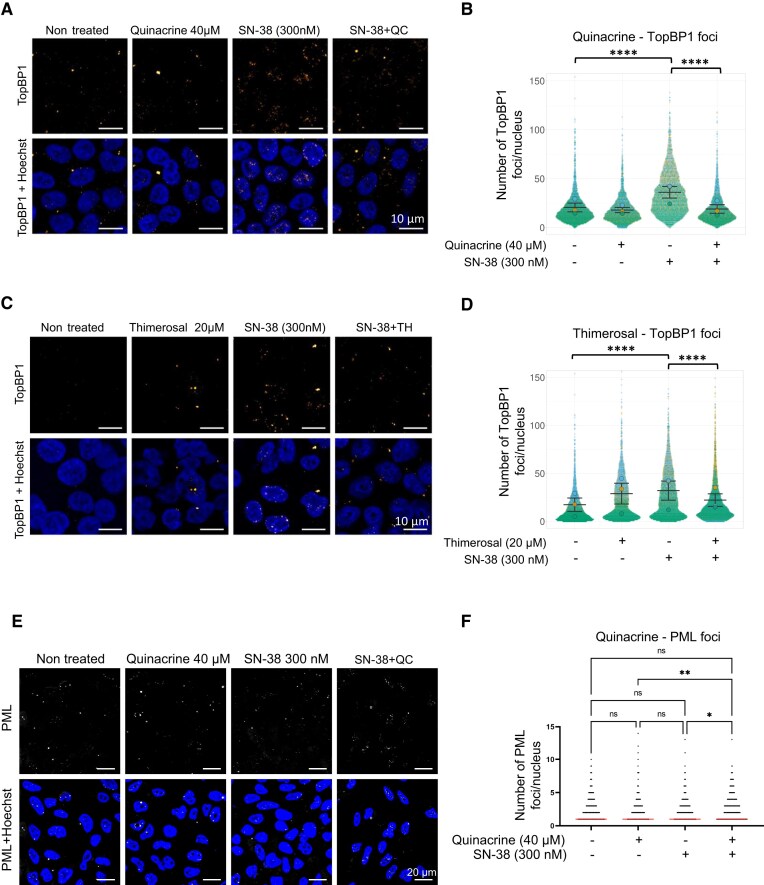
Quinacrine and thimerosal inhibit the formation of endogenous TopBP1 condensates. (**A**) Representative immunofluorescence images of TopBP1 foci in HCT116 cells incubated with quinacrine (40 µM) and/or SN-38 (300 nM) for 2 h, and (**B**) the corresponding quantification. (**C**) Representative immunofluorescence images of TopBP1 foci in HCT116 cells incubated with thimerosal (20 µM) and/or SN-38 (300 nM) for 2 h, and (**D**) the corresponding quantification. The experiment was repeated three times with similar results. Data from the three independent replicates were pooled and displayed as superplots in panels (B) and (D), error bars represent the standard error of the mean. (**E**) Representative immunofluorescence images of PML nuclear bodies in HCT116 cells incubated with quinacrine (40 µM) and/or SN-38 (300 nM) for 2 h and (**F**) the corresponding quantification. “−” denotes the absence of the indicated compound (control condition). CellProfiler 4.2.8 was used to quantify TopBP1 and PML nuclear foci (>300 cells analyzed per condition). Statistical significance was assessed using one-way ANOVA followed by Šídák’s post hoc test. ns, non-significant; *P-value <.05; **P-value <.01; and ****P-value <.0001. Scale bars, 10 µm for TopBP1 images and 20 µm for PML images.

This small-molecule screening approach implies that the hits may also interfere with the light-induced tetramerization of Cry2, which is critical for the nucleation of TopBP1 condensates. Therefore, to validate the two hits, we used complementary approaches. First, we investigated the effect of quinacrine and thimerosal on the condensation of endogenous TopBP1 in HCT116 cells. For this, we induced endogenous TopBP1 condensates by incubating cells with SN-38, the active metabolite of the topoisomerase I inhibitor irinotecan, for 2 h. We could visualize the formation of TopBP1 condensates as nuclear foci by immunofluorescence staining (Fig. 2A–D). In the presence of quinacrine or thimerosal, the number of TopBP1 foci induced by SN-38 was significantly reduced (Fig. 2B–D), consistent with the notion that these two small molecules inhibit TopBP1 condensation. To further explore the effect of quinacrine, we examined SN-38-resistant HCT116-SN50 cells. These cells exhibit elevated basal levels of TopBP1 foci that do not increase upon SN-38 treatment, in contrast to parental HCT116 cells [30] (Supplementary Fig. S2A). Nevertheless, quinacrine treatment, either alone or in combination with SN-38, significantly reduced TopBP1 condensates in these SN-38-resistant cells (Supplementary Fig. S2A). Moreover, mitotic TopBP1 condensates detected in HCT116 cells were not affected by treatment with SN-38, quinacrine, or their combination, indicating that the SN-38-induced TopBP1 foci are specific to interphase and predominantly reflect a replication stress-induced condensation event (Supplementary Fig. S1A). Conversely, quinacrine did not affect the formation of PML bodies, which are another nuclear biomolecular condensate type (Fig. 2E and F). Quinacrine (Supplementary Fig. S1B and C) and thimerosal (Supplementary Fig. S1D and E) inhibited SN-38-induced TopBP1 focus formation also in the prostate cancer cell line LNCaP. In this experimental condition of acute treatment with SN-38 for 2 h, high concentrations of quinacrine (40 µM) and thimerosal (20 µM) were required for optimal inhibition of TopBP1 foci. We chose this “high dose for a short time” condition to limit the confounding effects of prolonged incubation with chemotherapeutic agents and small-molecule inhibitors. In these experimental conditions, we did not observe major changes in cell cycle distribution (Supplementary Fig. S2B). Altogether, these data confirm the power of this screening approach to identify inhibitors of TopBP1 condensation.

### Quinacrine and thimerosal hinder ATR/Chk1 signaling in a human cell-free system

**Figure 3.**
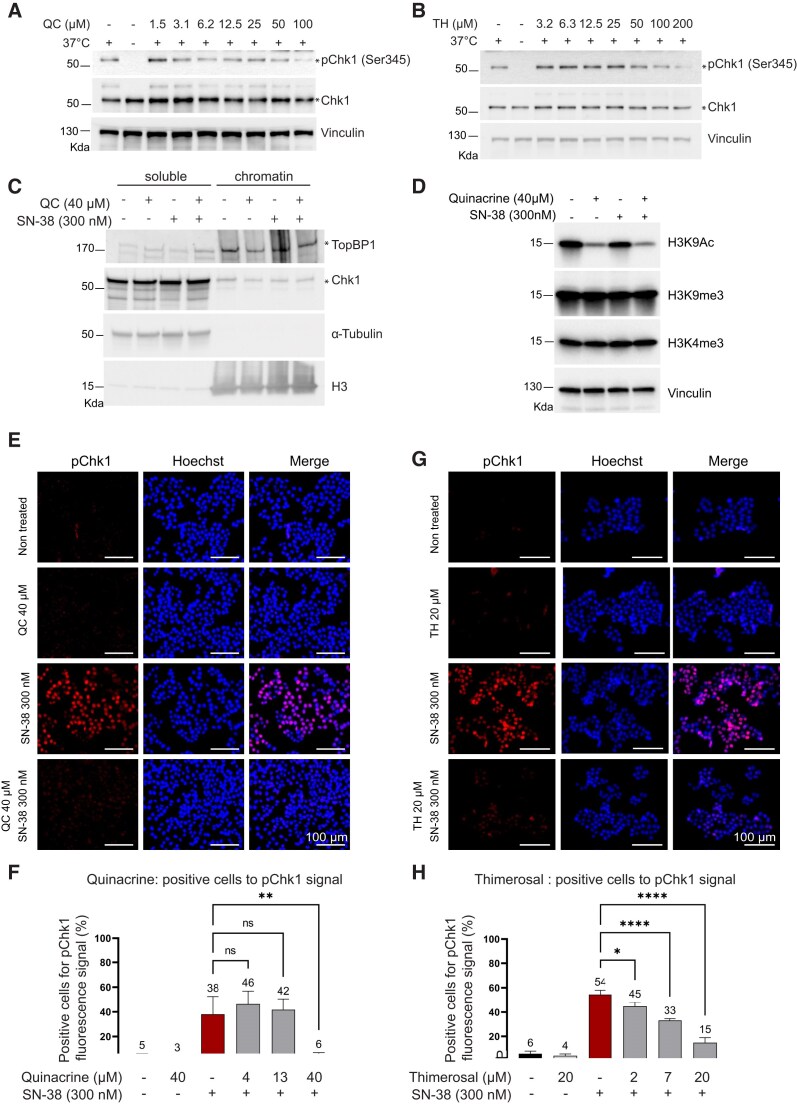
Quinacrine and thimerosal inhibit ATR/Chk1 signaling activation in cell-free extracts and in living cells. (**A**) Immunoblot showing phosphorylated Chk1 (pChk1) levels after incubation of nuclear extracts from optoTopBP1-HEK293 cells with increasing concentrations of quinacrine (QC) or (**B**) thimerosal (TH) for 30 min before induction of TopBP1 condensation at 37°C. The experiments were reproduced three times for QC in panel (A) and once for TH in panel (B). (**C**) Fractionation experiment to monitor TopBP1 association with chromatin in HCT116 cells incubated with quinacrine (40 µM) and/or SN-38 (300 nM) for 2 h. The experiments were reproduced three times. (**D**) Immunoblot showing the effect of SN-38 and/or quinacrine on histone marks in HCT116 cells. H3K4me3, H3K9Ac, and H3K9me3 levels were assessed to evaluate changes in chromatin state. Representative immunofluorescence images of pChk1 signals obtained with a Celigo Imaging Cytometer in HCT116 cells incubated with SN-38 (300 nM) and increasing concentrations of quinacrine (4.4, 13.3, and 40 µM) (**E**) and the corresponding quantification (**F**) or increasing concentrations of thimerosal (2.2, 6.7, and 20 µM) for 2 h (**G**) and the corresponding quantification (**H**). Error bars represent the standard deviation from three independent experiments; ns, non-significant; *P-value <.05; **P-value <.01; and ****P-value <.0001 (one-way ANOVA; SN-38 alone versus SN-38 with increasing doses of quinacrine or thimerosal).

TopBP1 condensation is essential for activating the ATR/Chk1 signaling pathway [21]. Thus, we investigated the effect of quinacrine and thimerosal on ATR using Chk1 phosphorylation as a marker of ATR activity. To exclude potential cytostatic and cytotoxic effects that may indirectly alter ATR signaling activation in live cells, we first examined the effect of quinacrine and thimerosal in a human cell-free system. We previously showed that Chk1, the master effector kinase of the ATR pathway, is promptly activated by phosphorylation in HeLa S3 cell nuclear extracts after incubation at 37°C for 10 min [21]. This reaction is dependent on endogenous ATR-activating DNA fragments that are present in the extracts and is inhibited by pre-incubation of the extract with ethidium bromide, which disrupts protein–DNA interactions. Likewise, pre-incubation of nuclear extracts with quinacrine and thimerosal inhibited the activation of endogenous ATR/Chk1 signaling in nuclear extracts of optoTopBP1-HEK293 cells (Fig. 3A and B, and Supplementary Fig. S3A and B).

### Quinacrine affects TopBP1 recruitment on chromatin

To explore the effect of quinacrine on the association of TopBP1 with chromatin in live cells, we fractionated HEK293 cells into soluble (nucleosolic) and chromatin-insoluble fractions. TopBP1 signal was increased in the soluble fraction of quinacrine-treated cells (Fig. 3C and Supplementary Fig. S3C), consistent with the notion that quinacrine alters the stability of the TopBP1–chromatin association. In agreement, TopBP1 signal intensity was decreased in the chromatin fraction of quinacrine-treated cells. In addition, we tested whether quinacrine treatment had an impact on chromatin topology by examining key histone modifications associated with chromatin accessibility. Immunoblot analysis of HCT116 cells revealed that quinacrine did not affect the levels of H3K4me3 or H3K9me3, which are associated with active promoters and constitutive heterochromatin, respectively (Fig. 3D). However, we observed a marked decrease in H3K9 acetylation (H3K9Ac), a histone modification linked to open and transcriptionally active chromatin (Fig. 3D). This reduction suggests that quinacrine promotes a more compact chromatin state, which may contribute to the impaired recruitment or retention of TopBP1 on chromatin. Together, these findings support the conclusion that quinacrine disrupts TopBP1–chromatin interactions, at least in part, by reducing chromatin accessibility.

### Quinacrine and thimerosal dampen ATR/Chk1 signaling in prostate and colorectal cancer cells

**Figure 4.**
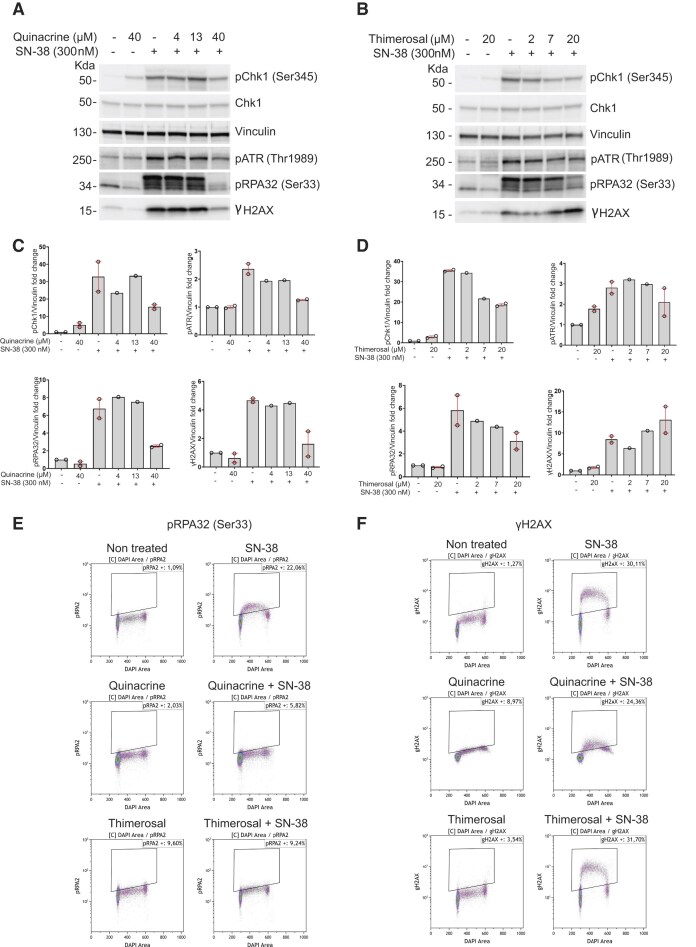
Quinacrine and thimerosal effects on RPA and H2AX phosphorylation. Immunoblots to assess the phosphorylation (p) level of the indicated ATR substrates in HCT116 cells incubated with SN-38 (300 nM) and/or quinacrine (4.4, 13.3, and 40 µM) (**A**) or thimerosal (2.2, 6.7, and 20 µM) (**B**) for 2 h and the corresponding quantifications (**C, D**) displayed as fold change over vinculin, normalized to non-treated cells. Bars represent mean ± standard error of the mean from two biological replicates. Two-dimensional flow cytometry analysis of pRPA32 (Ser33)/DAPI (**E**) and yH2AX (Ser139)/DAPI (**F**) fluorescence signals by flow cytometry after the extraction of soluble proteins from HCT116 cells, incubated with SN-38 (300 nM) and/or quinacrine (40 µM) or thimerosal (20 µM), as indicated.

Next, we used a Celigo Imaging Cytometer for high-throughput analysis of Chk1 phosphorylation on serine 345 (pChk1), as a marker of ATR activation, in HCT116 cells incubated with SN-38 for 2 h. SN-38 strongly increased the pChk1 fluorescence signal (Fig. 3E–H). Co-incubation with SN-38 and quinacrine (increasing concentrations) significantly decreased SN-38-induced pChk1 signal intensity in a dose-dependent manner (Fig. 3E and F), without major alterations in the cell cycle distribution (Supplementary Fig. S2B). We obtained similar results with thimerosal (Fig. 3G and H, and Supplementary Fig. S2B). In addition, we confirmed by immunoblotting that quinacrine and thimerosal inhibited SN-38-induced Chk1 phosphorylation (Fig. 4A–D). The two molecules inhibited the activation of the endogenous ATR/Chk1 signaling pathway also in LNCaP prostate cancer cells (Supplementary Fig. S4). Quinacrine and thimerosal also inhibited the phosphorylation of RPA32 on serine 33 (pRPA), but only quinacrine reduced the phosphorylation of H2AX on serine 139 (γH2AX) (both RPA32 and H2AX are ATR substrates) (Fig. 4A-D). This was confirmed by fluorescence-activated cell sorting after extraction of soluble proteins where pRPA32 fluorescent signal was increased in the S phase by 20% upon incubation with SN-38 and decreased by co-incubation with quinacrine or thimerosal (Fig. 4E and Supplementary Fig. S3D). Quinacrine strongly impaired the phosphorylation of H2AX in S/G2 cells (Fig. 4F and Supplementary Fig. S3E), whereas thimerosal had little impact on H2AX phosphorylation, suggesting a different mode of action on TopBP1 condensates (Fig. 4B, D, and F, and Supplementary Fig. S3E). We obtained similar results in LNCaP cells (Supplementary Fig. S5).

Collectively, these data suggest that quinacrine alters TopBP1 binding to the chromatin interface, which in turn inhibits the formation of damage-induced TopBP1 condensates that are required for activation of the ATR/Chk1 signaling pathway, and finally inhibits RPA and H2AX phosphorylation. Therefore, we focused on quinacrine.

### Interaction between quinacrine and conventional chemotherapy drugs

**Figure 5.**
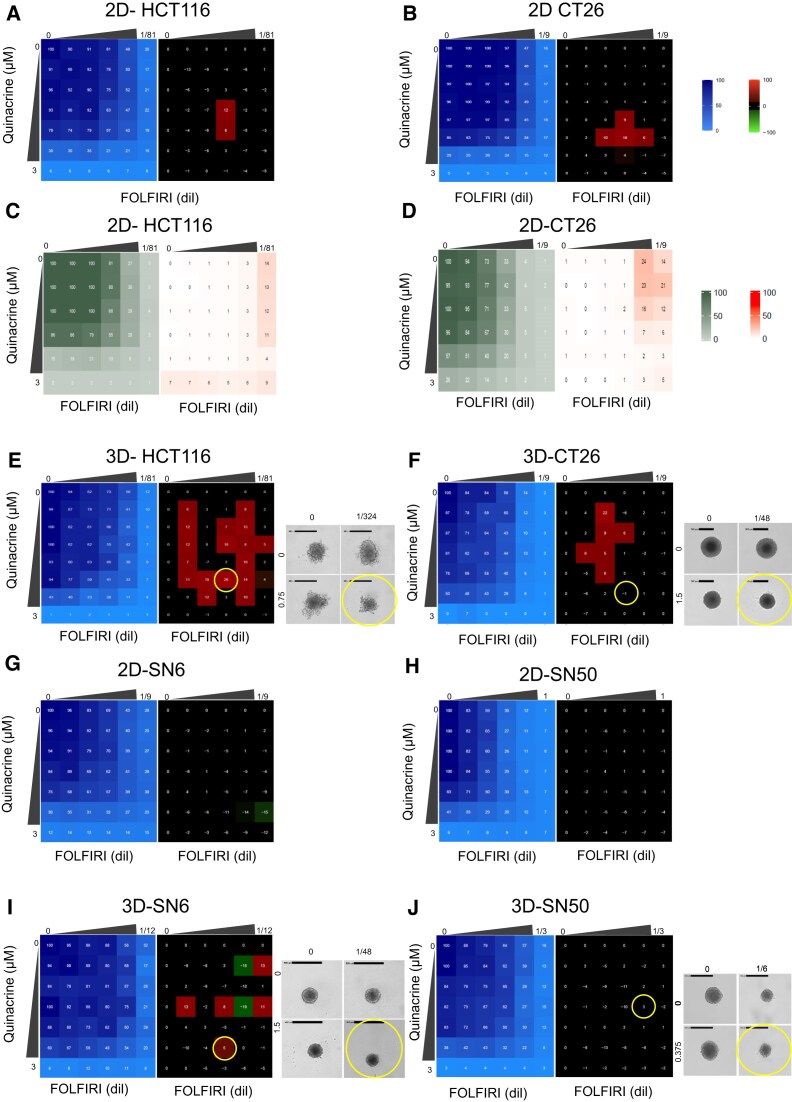
Quinacrine in combination with FOLFIRI displays additive and synergistic effects in 2D and 3D spheroid cell culture models (next page). Viability matrix (blue) and synergy matrix (red) of human HCT116 (**A**) and murine CT26 cells (**B**) incubated with increasing concentrations of quinacrine and FOLFIRI. FOLFIRI concentration for HCT116 cells: 5FU (from 0.009 to 0.148 µM), SN-38 (from 0.077 to 1.235 nM); and for CT26 cells: 5FU (0.083 to 1.333 µM), SN-38 (from 0.694 to 11.11 nM). Quinacrine concentration from 0.188 to 3 µM. Cell viability was assessed with the SRB assay (2D). Viability matrix (green) and cytotoxicity matrix (orange) of HCT116 (**C**) and CT26 (**D**) cells incubated with the same quinacrine and FOLIFRI concentrations; values were obtained with the “Dead + Total” cell viability application of the Celigo Imaging Cytometer (Nexcelom) using Hoechst/propidium iodide staining. Viability and synergy matrices of 3D spheroid models of HCT116 (**E**) and CT26 (**F**) cells (same quinacrine and FOLIFRI concentrations as before); viability was measured with the 3D-CellTiterGlo assay. Viability and synergy matrices of the SN-38-resistant HCT116-SN6 (SN6) (**G**) and HCT116-SN50 (SN50) (**H**) cell lines in 2D cultures; FOLFIRI: 5FU (from 0.083 to 1.133 µM and from 0.75 to 12 µM), SN-38 (from 0.694 to 11.11 nM and from 6.25 to 100 nM); quinacrine: from 0.188 to 3 µM (for both cell lines). Viability and synergy matrices of the SN-38-resistant HCT116-SN6 (SN6) (**I**) and HCT116-SN50 (SN50) (**J**) cell lines in 3D spheroid cultures; FOLFIRI: 5FU (from 0.0625 to 0.08 µM and from 0.25 to 4 µM), SN-38 (from 0.52 to 8.33 nM and from 2.08 to 33.33 nM); quinacrine: from 0.188 to 3 µM (for both cell lines). Cell viability was quantified with the 3D-CellTiterGlo assay. Representative brightfield images of spheroids were obtained with a Celigo imaging cytometer (Nexcelom). The synergy matrix was calculated as described in the “Materials and methods” section. Cells were incubated for 96 h (2D culture) and for 7 days (3D cultures).

Next, we analyzed the effect on cell survival of the combination of quinacrine and FOLFIRI, which contains irinotecan (SN-38 is the active metabolite of irinotecan) and 5-fluorouracil (5-FU). 5-FU is a pyrimidine analog that mainly inhibits thymidylate synthase (TS), disrupting DNA synthesis, and also incorporates into RNA and DNA, impairing their function [49]. First, we evaluated the interaction between quinacrine and 5-FU or SN-38 in HCT116 cells and in two SN-38-resistant colorectal cancer cell lines (HCT116-SN6 and HCT116-SN50), using a full-range concentration matrix approach and the SRB cytotoxicity assay. We quantified the percentage of cell growth (blue matrix) and the additive, synergistic, and antagonistic effects (black, red, and green matrices, respectively) (Supplementary Fig. S6). Quinacrine with 5-FU or SN-38 inhibited the survival of HCT116, HCT116-SN6, and HCT116-SN50 cells (blue matrices). These combinations also showed a major additivity effect and areas of synergistic interaction. Next, we assessed the interaction between quinacrine and FOLFIRI (FOLFIRI concentration range and IC50 are indicated in Supplementary Tables S2, S3, and S4) in HCT116 and CT26 (colorectal murine cell line) cells grown in 2D (Fig. 5A and B) and 3D spheroid cultures (Fig. 5E and F), which better recapitulates the structural complexity of solid tumors [50]. The quinacrine + FOLFIRI combination was mainly additive in 2D cultures and led to numerous focal areas of synergism in 3D cultures. In SN-38-resistant HCT116-SN6 and -SN50 cells, the combination was additive in 2D and 3D cultures (Fig. 5G–J). Thimerosal combined with 5-FU, SN-38, and FOLFIRI also had additive/synergistic effects in HCT116 cells (Supplementary Fig. S7). Then, to determine whether the quinacrine + FOLFIRI combination had an effect on cell death or proliferation, we performed propidium iodide/Hoechst labeling on the same full-range concentration matrix. We found that the combination was mainly cytostatic (Fig. 5C and D) in HCT116 cells and cytotoxic, at high FOLFIRI doses, in CT26 cells. In HCT116 cells, each drug alone and also FOLFIRI were mainly cytostatic, whereas in CT26 cells, quinacrine, FOLFIRI, 5-FU, and to a lesser extent SN-38 were also cytotoxic at high doses (Supplementary Fig. S6G and H). These results indicate that the quinacrine + FOLFIRI combination is mainly additive with focal synergistic areas in 3D culture and that this interaction is cytostatic in HCT116 cells and cytostatic and cytotoxic in CT26 cells.

### Quinacrine improves FOLFIRI effects in a mouse model of advanced colorectal cancer

**Figure 6.**
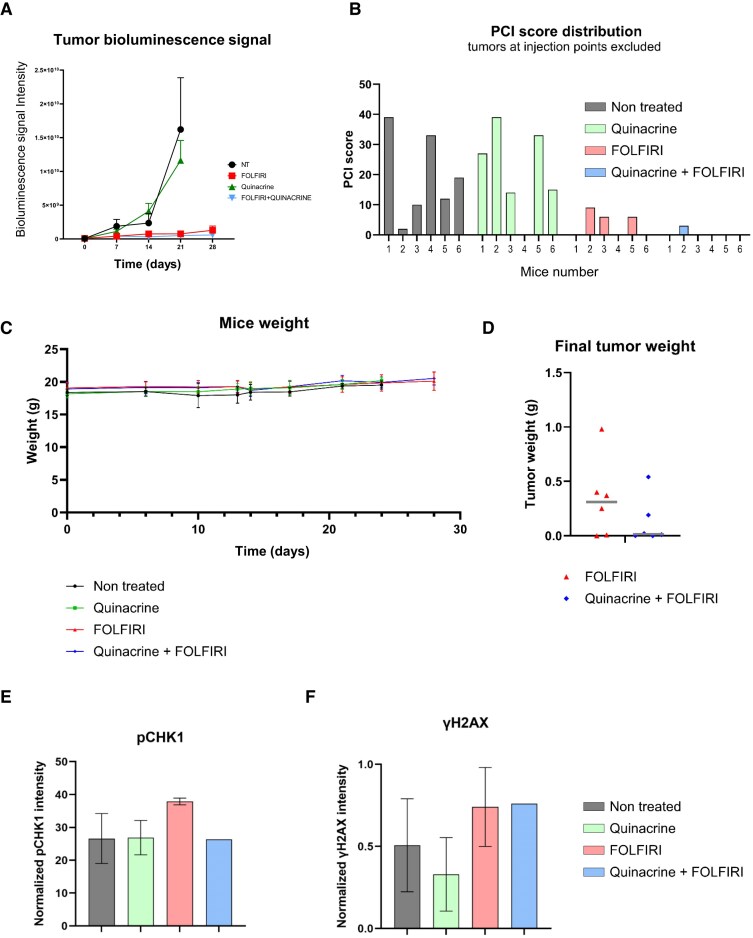
Quinacrine combined with FOLFIRI delays tumor growth and decreases the PCI in mice harboring CT26-luc cell grafts. (**A**) Tumor bioluminescence signal in control and treated mice (*n* = 6) during the treatment period. (**B**) PCI in the different treatment groups. At treatment end or when the critical threshold was reached, mice were dissected and the PCI score was calculated according to [41]. (**C**) Mean weight in the different groups during the treatment period. (**D**) Final tumor weight in the FOLFIRI and quinacrine + FOLFIRI groups. Quantification of pChk1 (Ser345) (**E**) and ɣH2AX (**F**) by immunofluorescence analysis of tumors collected at the end of treatment. Non-treated = 0.9% NaCl; quinacrine = 50 mg/kg quinacrine; FOLFIRI = 25 mg/kg 5-FU + 25 mg/kg irinotecan + 50 mg/kg leucovorin; quinacrine + FOLFIRI = quinacrine + FOLFIRI combination at the same concentrations as in the single-drug groups; *n* = 6 mice/group.

Lastly, to evaluate the therapeutic potential of the quinacrine + FOLFIRI combination *in vivo*, we used an orthotopic syngeneic graft model that mimics the peritoneal metastases from primary colorectal cancer. One week after the intraperitoneal (ip) graft of CT26-Luc cells (mouse CT26 colorectal cells that express luciferase), we divided the mice into four groups (*n* = 6): (i) non-treated (NT), (ii) FOLFIRI, (iii) quinacrine (50 µg/ml), and (iv) quinacrine (50 µg/ml) + FOLFIRI. We administered FOLFIRI by ip injection twice per week and quinacrine *per os* 3 days per week for 30 days. We monitored tumor growth by bioluminescence measurement during the treatment and by calculating PCI at the treatment end. Compared with the NT group, tumor growth was reduced in the FOLFIRI and the quinacrine + FOLFIRI groups (Fig. 6A), without any effect on the weight of mice (Fig. 6C). Moreover, the PCI values showed higher tumor regression in the quinacrine + FOLFIRI group than in the FOLFIRI group (Fig. 6B): 5/6 mice and 3/6 mice were completely cured in the quinacrine + FOLFIRI group and in the FOLFIRI group, respectively. The final tumor weight (at day 30) also was lower in the quinacrine + FOLFIRI group than in the FOLFIRI group (Fig. 6D). Moreover, immunofluorescence analysis of tumor samples collected at the treatment end showed that the pChk1 Ser345 signal was lower in the quinacrine + FOLFIRI group than in the FOLFIRI group (Fig. 6E), consistent with the results in HCT116 and LNCaP cells (Fig. 3 and Supplementary Fig. S4). Conversely, the γH2AX signal was similar in tumors from the quinacrine + FOLFIRI and FOLFIRI groups (Fig. 6F), unlike in the cell lines (Fig. 4 and Supplementary Fig. S5). This suggests that in mice treated with the quinacrine + FOLFIRI combination, DNA stress is maintained at high levels, while the S-phase checkpoint is dampened by the presence of quinacrine, providing a possible explanation for the synergistic effect of this combination. Overall, these findings indicate that the quinacrine + FOLFIRI combination has a stronger therapeutic effect on tumor growth and survival than FOLFIRI alone.

## Discussion

In this study, we used the high-throughput screening method described by Morano *et al.* [30] to identify molecules that block ATR/Chk1 signaling by inhibiting TopBP1 focus formation in colon cancer cells exposed to topoisomerase I inhibitors. Specifically, we screened the Prestwick library of 1520 FDA-approved drugs to validate this previously developed therapeutic strategy, which targets the assembly of biomolecular condensates essential for cancer cell responses to chemotherapeutic agents. Remarkably, approximately half of the hits were DNA-binding molecules or DNA intercalators, suggesting that they alter TopBP1 association with chromatin. TopBP1 forms globular clusters of nanometer size on chromatin [21]. Recent evidence suggests that chromatin provides a surface that promotes the condensation of nuclear scaffolds [44, 45]. Therefore, DNA-binding molecules may interfere with TopBP1 surface condensation on chromatin.

We screened the Prestwick Chemical Library of FDA-approved drugs as a mechanistically driven approach to drug repurposing. We focused on quinacrine, one of the best-studied drugs in terms of safety and pharmacokinetics [48]. Although it intercalates into DNA, quinacrine is not particularly toxic to normal cells and is well tolerated by animals and humans [31, 48]. The mechanism underlying quinacrine toxicity in cancer cells is still incompletely understood. Quinacrine induces p53 expression and inhibits the NF-kB pathway, possibly by trapping the p65 NF-kB subunit in an inactive state and by interfering with promoter binding [51, 52]. Alternatively, curaxins, and to a lesser extent quinacrine, are thought to inhibit NF-kB-dependent transcription by trapping the Facilitates Chromatin Transcription complex on chromatin [53]. Although quinacrine appears to function by altering the chromatin architecture, it does not induce DNA lesions and the DNA damage signaling pathways at a concentration (10 µM) higher than that required to inhibit NF-kB signaling [31, 53]. In addition, quinacrine synergizes with 5-FU in colon cancer [54], where it induces autophagy and apoptosis through activation of the p53/p21 axis [55].

The optogenetic screen and nuclear extract-based methods used in this study suggest that quinacrine inhibits ATR signaling directly by interfering with TopBP1 condensation. Although quinacrine targets multiple pathways, interference with TopBP1 condensate formation and DNA lesion signaling may represent a key mechanism of its preferential toxicity toward cancer cells. Furthermore, TopBP1 exerts various functions required for genome stability maintenance. Indeed, TopBP1 is involved in the initiation of DNA replication through its interaction with Treslin [56]. TopBP1 controls the repair of double-strand DNA breaks by homologous recombination via phosphorylation-regulated interactions with the anti-resection factor 53BP1 and the pro-resection factor BRCA1 [57]. In mitosis, TopBP1 forms filamentous structures and interacts with MDC1 to tether double-strand DNA breaks [58], and its interaction with TOP2A promotes the resolution of ultrafine anaphase bridges [59]. TopBP1 also promotes mitotic DNA synthesis through interaction with SLX4 [60, 61] and limits sister chromatid exchanges by interacting with Bloom’s syndrome protein [62]. TopBP1 also participates in the regulation of transcription, notably via interactions with E2F-1 and p53 to repress apoptosis [19, 63, 64]. Thus, beyond ATR signaling, disruption of TopBP1 compartmentalization by quinacrine may inhibit additional TopBP1-associated functions that determine the capacity of cancer cells to process DNA lesions in coordination with cell cycle progression.

## Supplementary Material

zcaf041_Supplemental_Files
