# Supplementary material for: Shining light on drug discovery: optogenetic screening for TopBP1 biomolecular condensate inhibitors
Source: NAR Cancer. 2025 Nov 3;7(4):zcaf041. doi: 10.1093/narcan/zcaf041 (PMC12582362; doi:10.1093/narcan/zcaf041)
Supplement: zcaf041_Supplemental_Files [file zcaf041_supplemental_files.zip › Supplementary data legends.docx]

**Supplementary data**

**Figure S1: Quinacrine and thimerosal inhibit TopBP1 condensate formation in a prostate cancer cell line.**

(**A**) Quantification showing the effect of SN-38 and/or quinacrine on interphase versus mitotic TopBP1 condensates. TopBP1 foci were scored using CellProfiler 4.2.8 and mitotic cells were identified based their specific Hoechst staining. The fold change of the number of TopBP1 foci per nucleus was normalized relative to Non treated cells. Bars represent mean ± standard error of the mean (SEM) from four independent experiments. Statistical significance was assessed using one-way ANOVA followed by Šídák’s post hoc tests. ns, non-significant, *p value <0.05, **p value <0.01, ***p value <0.001. (**B**) Representative immunofluorescence images of TopBP1 foci in LNCaP cells incubated or not with quinacrine (40 µM) and/or SN-38 (300 nM) for 2 h, and (**C**) the corresponding quantification. (**D**) Representative immunofluorescence images of TopBP1 foci in LNCaP cells incubated or not with thimerosal (20 µM) and/or SN-38 (300 nM), and (**E**) the corresponding quantification. The experiment was repeated thrice, and similar results were obtained. Cell profiler was used for the quantification of TopBP1 foci. ****p value <0.0001 (Mann-Whitney test). Scale bars, 10 µm.

**Figure S2: Impact of quinacrine and thimerosal in SN-38-resistant HCT116 cells and the cell cycle. (A)** **Quantification of the effect of SN-38 and/or quinacrine on TopBP1 foci in parental HCT116 and SN-38–resistant HCT116-SN50 cells.** The experiment was repeated three times with similar results; data were pooled and displayed as superplot. Bars represent the mean ± SEM from three independent experiments. Statistical significance was assessed using one-way ANOVA followed by Šídák’s post hoc test. ns, non-significant, ****p value <0.0001. **(B)** Cell cycle analysis using propidium iodide staining after incubation with 40 µM of quinacrine or 20 µM of thimerosal for 2 h.

**Figure S3: Quantification of Figures 3A, B and 3C, 4E and 4F.**

(**A**) Fold change (normalized to Non treated, 37 °C), of the pChk1/Chk1 band intensity ratio upon increasing Quinacrine concentrations in nuclear extracts, quantified with Fiji, corresponding to Figure 3A (n=3). (**B**) Fold change (normalized to Non treated, 37 °C), of the pChk1/Chk1 band intensity ratio upon increasing Thimerosal concentrations in nuclear extracts, quantified with Fiji, corresponding to Figure 3B (n=1). (**C**) Fold change (normalized to Non treated) of the Chromatin-bound (C) TopBP1 faction over the Soluble (S) TopBP1 faction. Band intensities were assessed using Fiji, corresponding to Figure 3C (n=3). (**D–E**) Quantification of the percentage of pRPA32(Ser33) (D) or γ-H2AX (E) positive cells, corresponding to Figures 4E and 4F, respectively. 20,000 cells were analyzed per experiment using the Kaluza analysis software. Experiments were repeated 2-6 times, as indicated by the number of dots in the dot plots. Bars represent the mean ± SEM from 2-6 independent replicates. Statistical significance was assessed using one-way ANOVA followed by Šídák’s post hoc tests. ns, non-significant, ***p<0.001, ****p<0.0001.

**Figure S4: Quinacrine and thimerosal inhibit ATR/Chk1 signaling activation in the LNCaP prostate cancer cell line.**

(**A-B**) Immunoblots showing pChk1 signals in LNCaP cells incubated with SN-38 (300 nM) and/or quinacrine (40 µM) (A) and/or thimerosal (2.2 µM, 6.7 µM, 20 µM) (B) after 2 h of treatment.

**Figure S5: Impact of quinacrine and thimerosal on RPA and H2AX phosphorylation in the LNCaP prostate cancer cell line.**

(**A**) Immunoblots showing the phosphorylation of the indicated ATR substrates in LNCaP cells incubated with SN-38 (300 nM) and/or quinacrine (40 µM) or thimerosal (20 µM) for 2 h. (**B-C**) Two dimensional pRPA32 (Ser33)/DAPI (B) and yH2AX (Ser139)/DAPI analysis by fluorescence activated cell sorting after extraction of soluble proteins from LNCaP cells incubated with SN-38 (300 nM) and/or quinacrine (40 µM) (B) or thimerosal (20 µM) (C).

**Figure S6: Effects of quinacrine with SN-38 and 5-FU in 2D cultures of human and murine colorectal cancer cell lines and in SN-38-resistant human colorectal cancer cell lines.**

(**A-B**) Viability matrix (blue) and synergy matrix (red) of human HCT116 cells incubated with quinacrine (from 0.188 µM to 3 µM) and 5-FU (from 0.3125 to 5 µM) (A) or SN-38 (from 0.25 to 4 nM) (B). (**C-D**) Viability matrix (blue) and synergy matrix (red) of SN-38-resistant HCT116-SN6 cells (6 times more than wild type) incubated with quinacrine (from 0.188 µM to 3 µM) and 5-FU (from 0.625 µM to 10 µM) (C) or SN-38 (from 0.625 nM to 10 nM) (D). (**E-F**) Viability matrix (blue) and synergy matrix (red) of SN-38-resistant HCT116-SN50 cells (50 times more than wild type) incubated with quinacrine (from 0.188 µM to 3 µM) and 5-FU (from 0.625 µM to 10 µM) (E) or SN-38 (from 6 nM to 100 nM) (F). (**G-H**) Growth inhibition and cytotoxicity in HCT116 (G) and CT26 (H) cells incubated with quinacrine, FOLFIRI, SN-38 or 5-FU. In all experiments, cells were incubated for 96 h. What are the red and green curves?

**Figure S7: Effect of thimerosal combined with SN-38, 5-FU, or FOLFIRI in 3D spheroids of HCT116 cells.**

(**A**) Viability matrix (blue) and synergy matrix (red) (left panels) of HCT116 cells incubated with increasing concentrations of thimerosal and SN-38 for 72 h. Representative brightfield images of spheroids incubated with the indicated concentrations of thimerosal and SN-38 (right panels); pink squares highlight the synergistic effect of the combination. Propidium iodide was added to visualize dead cells. Cell viability was assessed with the CTG assay. (**B**) Left panel shows a representative brightfield image of the plate, and the right panel is a red fluorescent image of the plate. (**C-D**) Same as in (A) and (B) respectively, but thimerosal was combined with 5-fluorouracil. (**E**) Same as in A, but thimerosal was combined with FOLFIRI.

**Table S1: Positive hits identified in the optogenetic screen.** This table provides detailed information on the identified molecules, including chemical names, mean z-score (calculated based on the molecule ability to inhibit TopBP1 foci from three replicates), therapeutic class and effect, and the names of the target proteins. #N/A: Not Available**.**

**Table S2:** FOLFIRI concentration range used in the indicated cell lines.

**Table S3:** IC50 in function of the cell line for FOLFIRI, 5-FU, SN-38 and quinacrine based on the 2D cell culture data.

**Table S4:** IC50 in function of the cell line for FOLFIRI, 5-FU, SN-38 and quinacrine based on the 3D spheroid data.

**Table S5**: Full list of compounds screened in the Prestwick library with replicate counts, individual scores, average score, and hit assignment
