# Supplementary material for: Shining light on drug discovery: optogenetic screening for TopBP1 biomolecular condensate inhibitors
Source: NAR Cancer. 2025 Nov 3;7(4):zcaf041. doi: 10.1093/narcan/zcaf041 (PMC12582362; doi:10.1093/narcan/zcaf041)
Supplement: zcaf041_Supplemental_Files [file zcaf041_supplemental_files.zip › Supp_tables-2-4.pdf]

		FOLFIRI concentration range				
**HCT116**	FOLFIRI(dilution factor)	**1296**	**648**	**324**	**162**	**81**
	5FU (µM)	0.009	0.019	0.037	0.074	0.148
	SN38 (nM)	0.077	0.154	0.309	0.617	1.235
**CT26**	FOLFIRI(dilution factor)	**144**	**72**	**36**	**18**	**9**
	5FU (µM)	0.083	0.167	0.333	0.667	1.333
	SN38 (nM)	0.694	1.389	2.778	5.556	11.11
**HCT116-SN6**	FOLFIRI(dilution factor)	**144**	**72**	**36**	**18**	**9**
	5FU (µM)	0.083	0.167	0.333	0.667	1.333
	SN38 (nM)	0.694	1.389	2.778	5.556	11.11
**HCT116-SN50**	FOLFIRI(dilution factor)	**16**	**8**	**4**	**2**	**1**
	5FU (µM)	0.750	1.500	3.000	6.000	12.00
	SN38 (nM)	6.250	12.50	25.00	50.00	100.0
**HCT116**	5FU (µM)	**0.3125**	**0.625**	**1.25**	**2.5**	**5**
	SN38 (nM)	**0.25**	**0.5**	**1**	**2**	**4**
**HCT116-SN6**	5FU (µM)	**0.625**	**1.25**	**2.5**	**5**	**10**
	SN38 (nM)	**0.625**	**1.25**	**2.5**	**5**	**10**
**HCT116-SN50**	5FU (µM)	**0.625**	**1.25**	**2.5**	**5**	**10**
	SN38 (nM)	**6**	**13**	**25**	**50**	**100**
**All cell lines**	Quinacrine (µM)	**0.188**	**0.375**	**0.75**	**1.5**	**3**

Table S2

		IC50 2D-culture			
		**IC50 FOLFIRI**	**IC50 5-FU (µM)**	**IC50 SN38 (nM)**	**IC50 Quinacrine (µM)**
**HCT116**	**FOLFIRI (facteur de dil)**	**0.0055 ± 0.00073**			
	5FU (µM)	0.066	2.47 ± 0.53		
	SN38 (nM)	0.55		0.73 ± 0.15	
					1.31 ± 0.17
**CT26**	**FOLFIRI (facteur de dil)**	**0.041 ± 0.007**			
	5FU (µM)	0.492	0.66 ± 0.09		
	SN38 (nM)	4.1		6.13 ± 0.57	
					1 ± 0.08
**HCT116-SN6(2D9)**	**FOLFIRI (facteur de dil)**	**0.039 ± 0.0048**			
	5FU (µM)	0.468	2.55 ± 0.45		
	SN38 (nM)	3.9		4.9 ± 1.5	
					1.18 ± 0.06
**HCT116-SN50 (C4)**	**FOLFIRI (facteur de dil)**	**0.175 ± 0.025**			
	5FU (µM)	2.1	3.2 ± 0.6		
	SN38 (nM)	17.5		47 ± 7	
					1.27 ± 0.1

Table S3

IC50 3D-culture					
		**IC50 FOLFIRI**	**IC50 5-FU**	**IC50 SN38**	**IC50 Quinacrine**
**HCT116**	**Folfiri (facteur de dil)**	**0.004 ± 0.0005**			
	5FU (µM)	0.048	0.85 ± 0.25		
	SN38 (nM)	0.4		0.66 ± 0.11	
					1.57 ± 0.3
**CT26**	Folfiri (facteur de dil)	**0.033 ± 0.0022**			
	5FU (µM)	0.396	0.6		
	SN38 (nM)	3.3		6.6	
					1.41 ± 0.15
**HCT116-SN6(2D9)**	**Folfiri (facteur de dil)**	**0.0285 ± 0.0005**			
	5FU (µM)		1.4 ± 0.1		
	SN38 (nM)			2.15 ± 0.45	
					1.42 ± 0.135
**HCT116-SN50 (C4)**	**Folfiri (facteur de dil)**	**0.175 ± 0.025**			
	5FU (µM)		1.33 ± 0.125		
	SN38 (nM)			36.5 ± 11.5	
					1.49 ± 0.178

Table S4
