# Supplementary material for: Shining light on drug discovery: optogenetic screening for TopBP1 biomolecular condensate inhibitors
Source: NAR Cancer. 2025 Nov 3;7(4):zcaf041. doi: 10.1093/narcan/zcaf041 (PMC12582362; doi:10.1093/narcan/zcaf041)
Supplement: zcaf041_Supplemental_Files [file zcaf041_supplemental_files.zip › Table S1.pdf]

Chemical name	ore_avera	Therapeutic class	Therapeutic effect	Target name	Literature reference
Tolonium chloride	-9,7	Dermatology	Cardiovascular	DNA	J Oral Maxillofac Pathol. 2012 May;16(2):251-5
Chlorquinaldol	-9,1	Infectiology	Antibacterial	Bacteria Growth	Life sciences, vol. 82, nos 3-4, 2008, p. 210-217
Thimerosal	-8,2	Infectionlogy	Antiseptic	inositol 1,4,5-trisphosphate-sensitive calcium-release channel	Am J Syph Gonorrhea Vener Dis. 1946 Jul;30:34
Methylene blue	-8,0	Dermatology	Antiseptic	NADH-cytochrome b5 reductase 3	Antioxid Redox Signal. 2012 Aug 15;17(4):544-54
Proflavine hemisulfate	-6,9	Infectiology	Antiseptic	Bacterial RNA	Br Med J. 1917 Jul 21;2(2951):70-5
Auranofin	-4,7	Metabolism	Analgesic	#N/A	Clin Rheum Dis. 1984 Aug 10(2):369-83
Pyrvinium pamoate	-4,7	Metabolism	#N/A	#N/A	Antimicrob Agents Chemother. 2008 Sep 52(9):3106-12
Verteporfin	-4,7	Ophthalmology	#N/A	#N/A	Clin Ophthalmol. 2013 7:1867-1875
Disulfiram	-4,4	Metabolism	Antabuse effect	Alcohol dehydrogenase	Alcohol Clin Exp Res. 2011 Oct 35(10):1749-58
Propidium iodide	-3,6	Infectiology	Antibacterial	#N/A	Methods Mol Biol. 2007 399:15-29
Thiostrepton	-3,3	Infectiology	Antibacterial	#N/A	PLoS One. 2011 Feb 18 6(2):e17110
Quinacrine dihydrochloride hydrate	-3,1	Infectiology	Antihelmintic	Monoamines oxidases	SEATROPH. 1980 Jun 11(2):280-4
Epirubicin hydrochloride	-3,0	Oncology	Antineoplastic	#N/A	Breast. 2012 Apr 21(2):142-9
Oxibendazol	-3,0	Metabolism	#N/A	#N/A	Am J Vet Res. 1976 Nov 37(11):1285-6
1,8-Dihydroxyanthraquinone	-3,0	Gastroenterology	Laxative	Serotoninergic 5-HT3 receptor	Carcinogenesis. 1997 Jun 18(6):1259-63
Protriptyline hydrochloride	-2,9	Central Nervous System	Antidepressant	Adrenergic uptake	Drugs. 2004 64(13):1385-99
Posaconazole	-2,5	Infectiology	Antifungal	#N/A	N Engl J Med. 2007 Jan 25;356(4):348-59
Digoxigenin	-2,5	Diagnostic	#N/A	#N/A	Mol Biotechnol. 1997 Apr 7(2):121-4
Thonzonium bromide	-2,5	Dermatology	Antiseptic	#N/A	Pharm Res. 1986 Oct 3(5):298-301
Triclabendazole	-2,4	Infectiology	Antihelmintic	Tubuline	J Helminthol. 2009 Jun 83(2):139-50
Podophyllotoxin	-2,4	Metabolism	Antiviral	#N/A	Curr Med Chem. 2009 16(3):327-49
Digoxin	-2,4	Cardiovascular	Cardiotonic	Na+ K+ ATPase	Int J Cardiol. 2013 Apr 15 164(3):365-7
Gemcitabine	-2,3	Oncology	Antineoplastic	#N/A	Curr Med Chem. 2012 19(7):1076-87
Topotecan	-2,3	Oncology	Antineoplastic	#N/A	Oncology. 1999 56(1):1-12
Monobenzone	-2,3	Dermatology	#N/A	#N/A	Pigment Cell Melanoma Res. 2011 Aug 24(4):673-9
Florfenicol	-2,3	Metabolism	Antibacterial	50S unit	FEMS Microbiol Rev. 2004 Nov 28(5):519-42
Mibefradil	-2,1	Central Nervous System	Antihypertensive	Voltage-gated L-type calcium channel alpha-1C subunit	Mol. Pharmacol. (1995) 48 (3): 540-9
Fendiline hydrochloride	-2,1	Cardiovascular	Antianginal	Ca2+ channel	Pharmatherapeutica. 1987 5(2):103-36
Digitoxigenin	-2,1	Cardiovascular	Cardiotonic	Na+ K+ ATPase	Eur J Clin Pharmacol. 1985 28(6):649-52
Ancitabine hydrochloride	-2,0	Oncology	Antineoplastic	DNA	J Pharm Sci. 1984 Jul;73(7):896-902
Flubendazol	-2,0	Metabolism	#N/A	#N/A	Am J Trop Med Hyg. 1984 Jul 33(4):627-31
