# Supplementary figures and images for: Shining light on drug discovery: optogenetic screening for TopBP1 biomolecular condensate inhibitors
Source: NAR Cancer. 2025 Nov 3;7(4):zcaf041. doi: 10.1093/narcan/zcaf041 (PMC12582362; doi:10.1093/narcan/zcaf041)

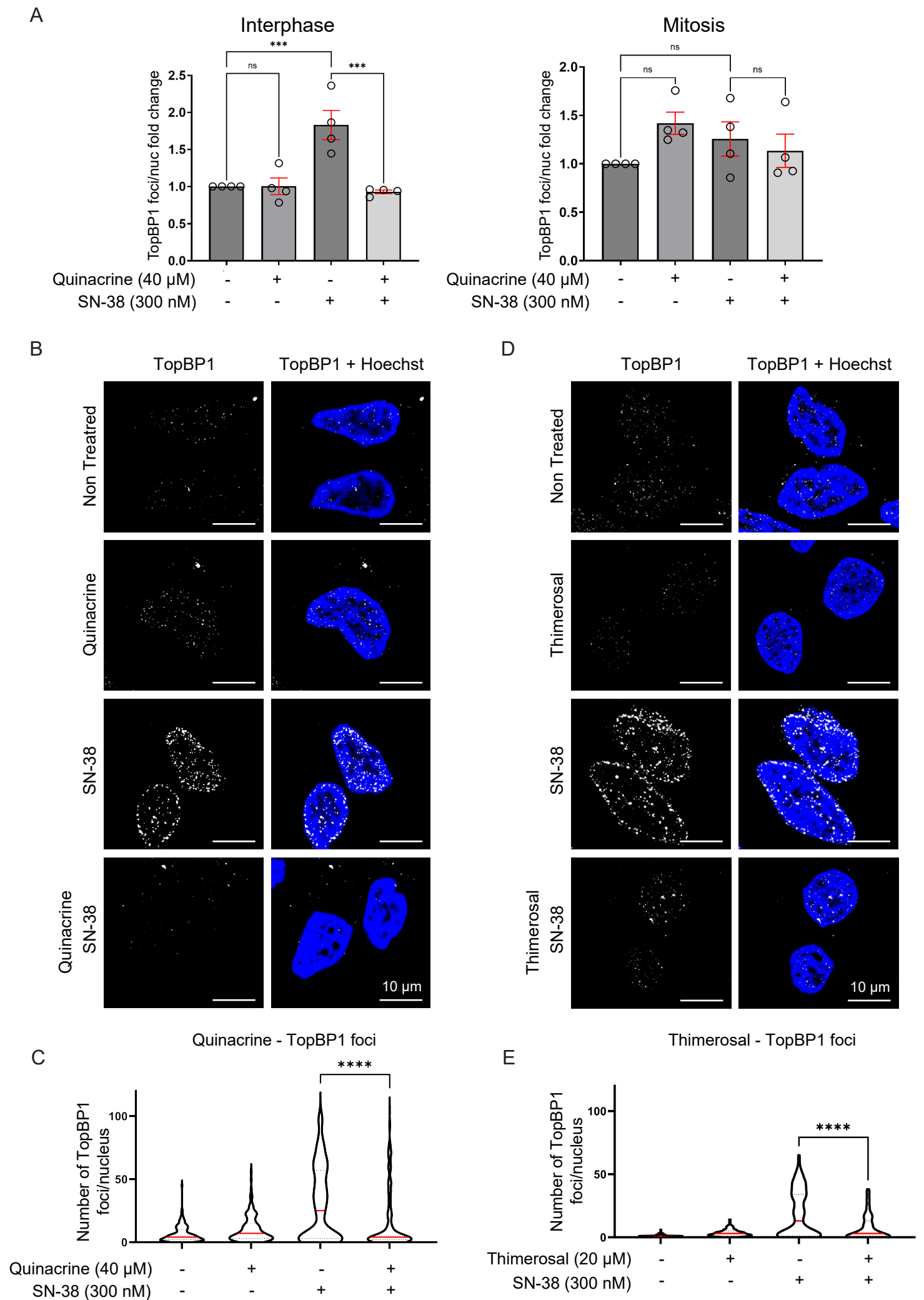

Figure S1

A

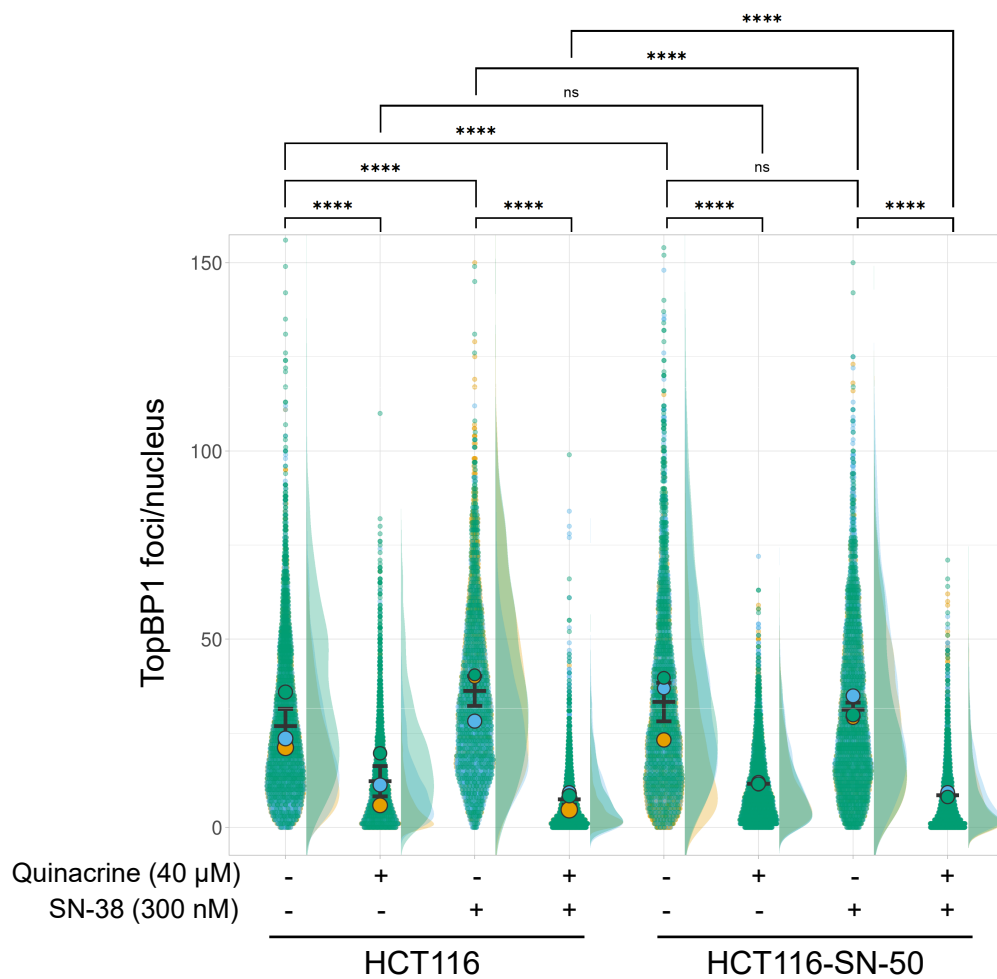

B

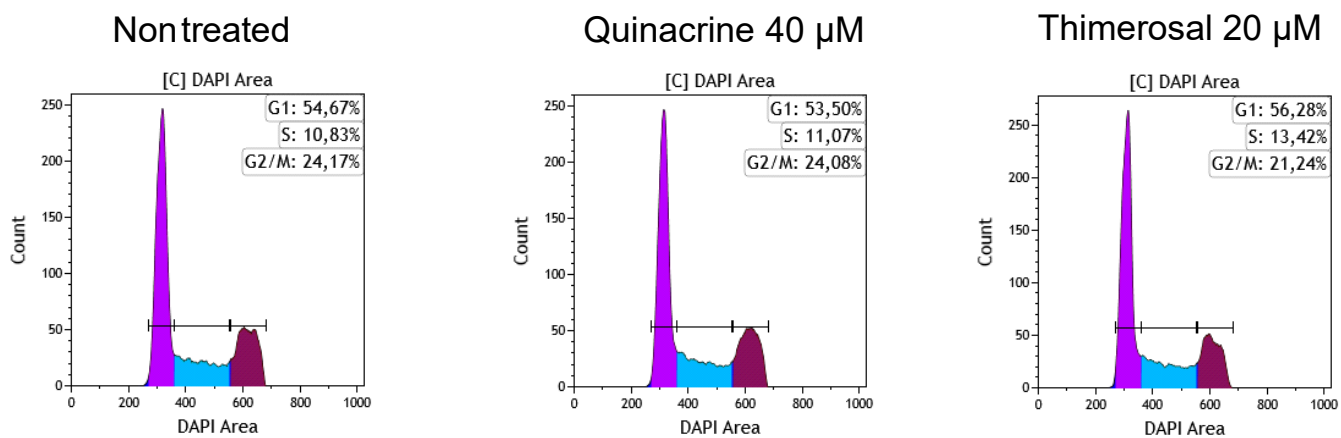

Figure S2

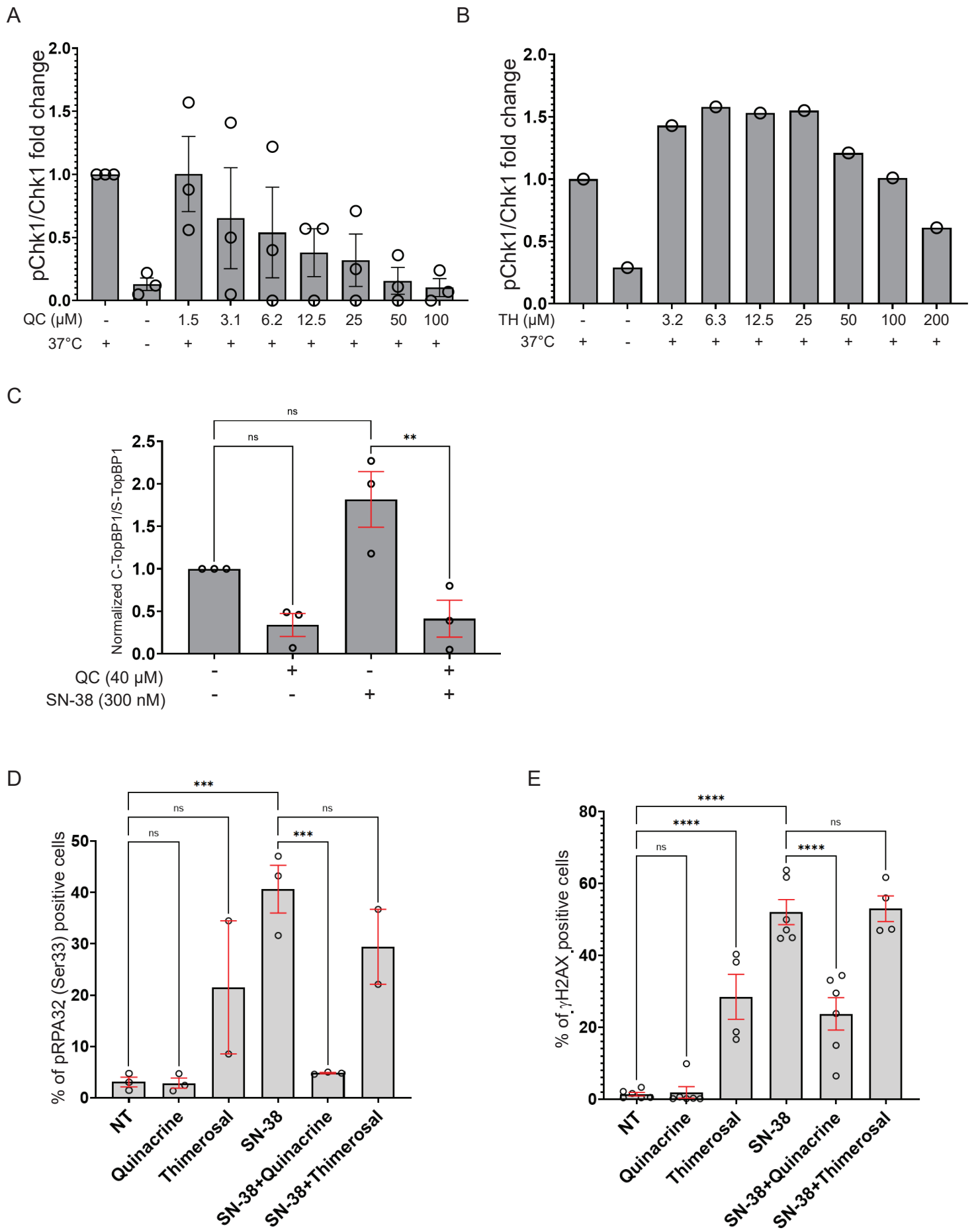

Figure S3

A

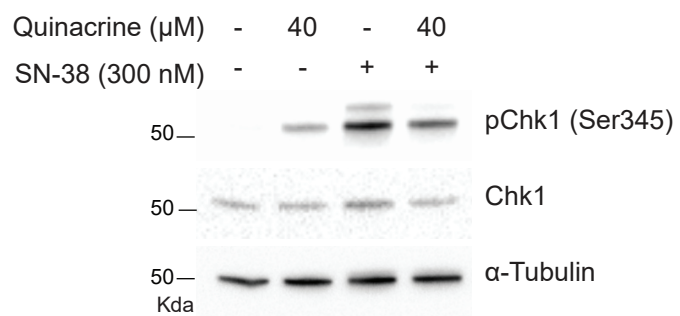

B

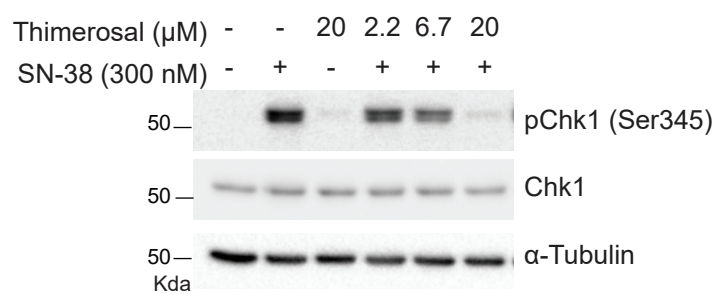

Figure S4

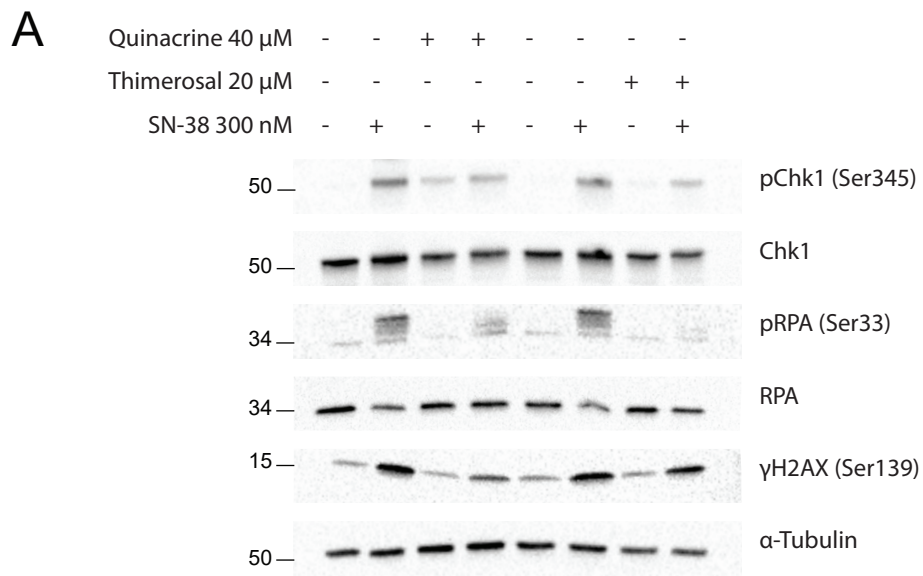

**B**

pRPA32 (Ser33)

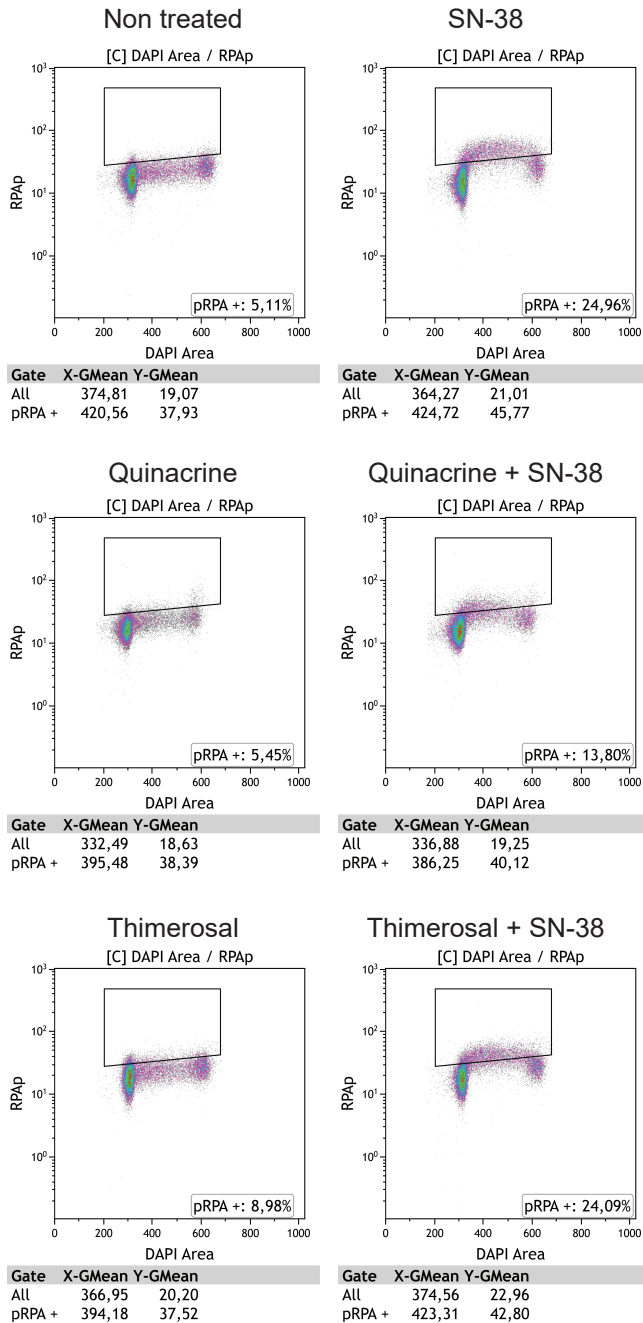

**C**

γH2AX

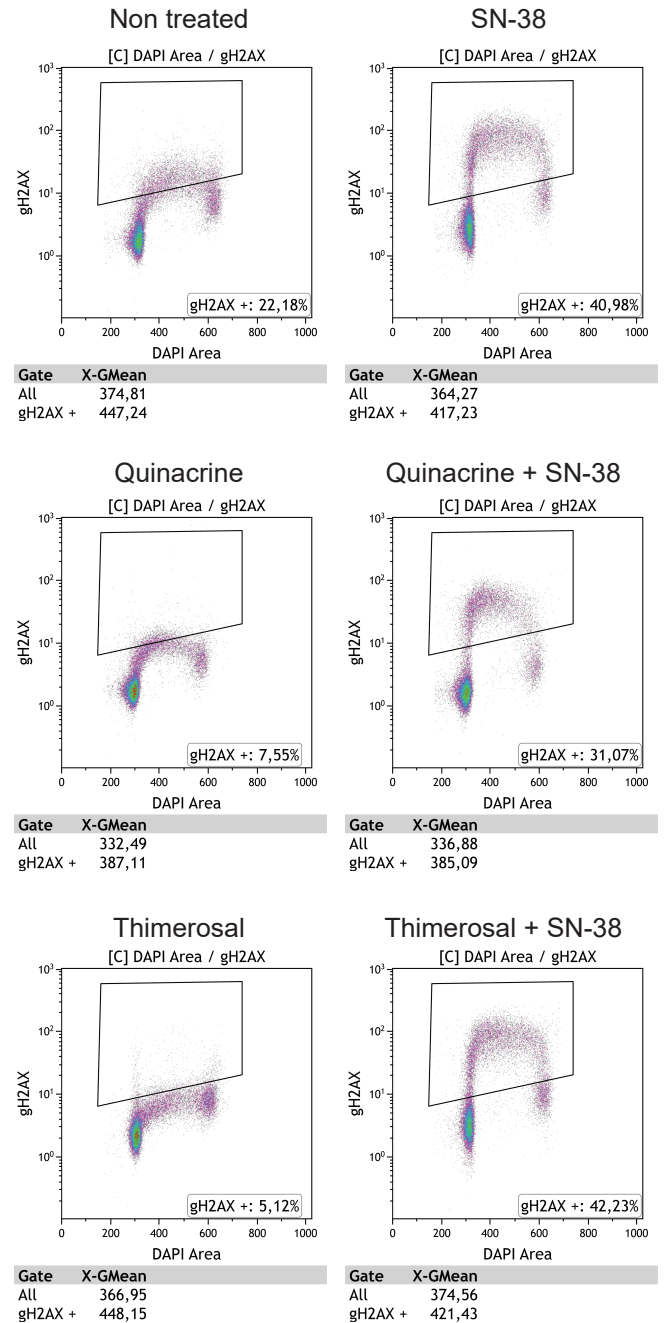

Figure S5

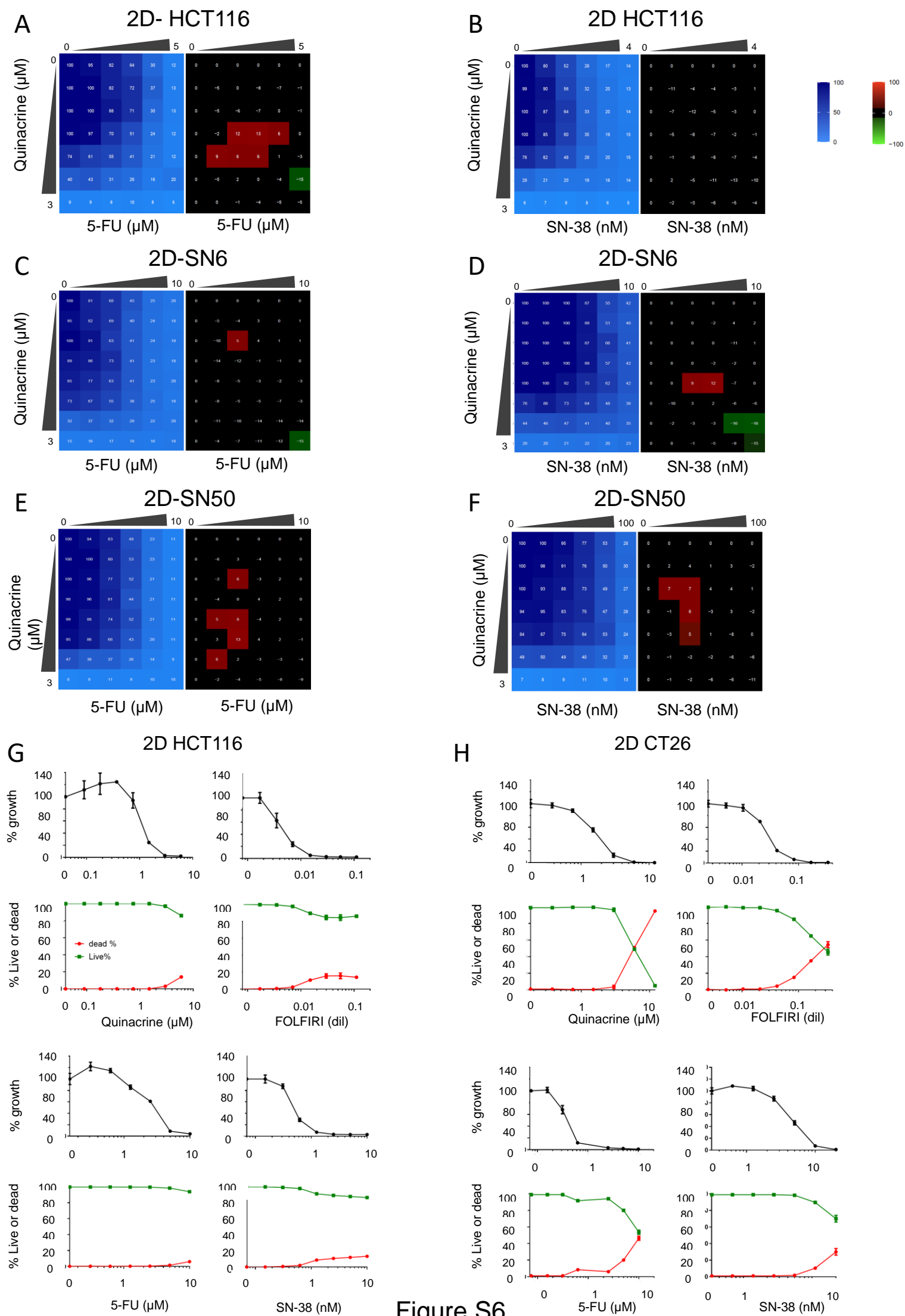

Figure S6

Supplement: zcaf041_Supplemental_Files [file zcaf041_supplemental_files.zip › Supp_Figures 1-7.pdf]
